# Effects of exercise on metabolic risk, cardiovascular fitness, and body composition in elderly women of the past decade: a systematic review and meta-analysis

**DOI:** 10.1080/15502783.2026.2675444

**Published:** 2026-06-02

**Authors:** Jing Shen, Jin Peng, YuJie Luan, XinYi Liu, Jian Li, Jinhui Wu

**Affiliations:** a Center of Gerontology and Geriatrics, National Clinical Research Center for Geriatrics, West China Hospital, Sichuan University, Chengdu, China; b Sports Medicine Center, Department of Orthopedic Surgery/Orthopedic Research Institute, West China Hospital, Sichuan University, Chengdu, China

**Keywords:** Elderly women, exercise, metabolic risk, cardiovascular health, body composition

## Abstract

**Background:**

Age-related reductions in physical activity and unfavorable body composition changes promote metabolic dysfunction and cardiovascular risk elevation in older populations. Due to estrogen-related factors, differences in cardiovascular risks, and musculoskeletal conditions, exercise may be one of the most accessible and widely applicable lifestyle interventions for older women. A comprehensive and systematic search has not yet been carried out on the effects of exercise on cardiovascular risk and its related indicators in elderly women. This meta-analysis evaluates exercise effects on metabolic risk, cardiovascular health, and body composition in healthy elderly women.

**Methods:**

Following PRISMA guidelines, we systematically searched PubMed, Cochrane Library, Web of Science, Embase, Scopus, CNKI, VIP, Wanfang, and Sinomed (2014–2024) for randomized controlled trials (RCTs) comparing supervised exercise with nonexercise controls. Data were analyzed with fixed- and random-effect models in Stata 17.0. The Cochrane RoB2 tool assessed bias risk, while the certainty of evidence was evaluated through the GRADE approach.

**Results:**

Twenty-three RCTs (33 intervention arms, 23 controls) were included. Exercise significantly reduced triglycerides (TG) (−8.56 mg/dL, 95% CI: −16.72, −0.40), total cholesterol (TC)(−26.67 mg/dL, 95% CI: −34.92, −18.42), low-density lipoprotein cholesterol (LDL-C) (−23.77 mg/dL, 95% CI: −34.48, −13.05), blood glucose (Glu) (−5.59 mg/dL, 95% CI: −10.12, −1.06), and C-reactive protein (CRP) (−0.86 mg/L, 95% CI: −1.37, −0.35). Cardiovascular improvements included increased VO2peak (+2.78 mL/kg/min, 95% CI: 1.87, 3.70) and reduced systolic blood pressure (SBP) (−8.35 mmHg) and diastolic blood pressure (DBP) (−3.26 mmHg). Regarding body composition, relative body fat (RF, i.e. body fat percentage) decreased (−2.47%, 95% CI: −3.42, −1.53), but no significant changes were observed in body weight, trunk fat mass (TFM), waist circumference (WC), fat-free mass (FFM), or skeletal muscle mass (SMM). According to the GRADE framework, the evidence was of moderate certainty for reductions in TC, TG, RF, VO₂peak, and SBP; and low certainty for all other outcomes.

**Conclusion:**

This meta-analysis provides evidence that appropriate exercise (aerobic, resistance, combined exercise) effectively reduces cardiovascular risk factors in elderly women via dual mechanisms of body composition remodeling and metabolic homeostasis enhancement, with particular efficacy in lipid regulation and blood pressure control.

## 1. Introduction

Epidemiological studies suggest that, projected by 2050, 22% of the world's population will be aged over 60 [1]. With aging, physical activity levels generally decrease in older adults, resulting in adverse changes in body composition, such as reduced muscle mass and increased fat mass [2,3]. These changes not only exacerbate metabolic dysfunction but also increase the risk of cardiovascular disease. In this context, exercise, as a nonpharmacological intervention, has great value in improving the health of the elderly [4–6].

The narrow definition of “exercise” is a planned or structured physical activity that can be aerobic exercise, resistance training, or combined with aerobic and resistance training [7]. Resistance training intensity is usually prescribed using the maximum number of repetitions (RM) method, which is characterized by causing neuromuscular fatigue within a specified interval (e.g. 10, 12, or 15 RM) [8,9]. Aerobic exercise includes endurance exercises like walking, jogging, and cycling [7]. While their physiological mechanisms differ—with aerobic training primarily enhancing cardiovascular function by improving oxygen utilization, and mitochondrial density [10,11], and resistance training stimulating neuromuscular adaptations, muscle protein synthesis to increase metabolic rate [12,13]—all these forms confer significant cardiometabolic benefits, making them relevant components of a comprehensive exercise intervention for older adults [14].

Studies have reported that moderate-intensity walking is an easily accessible daily exercise and an effective nonpharmacological treatment for reducing obesity and cardiovascular disease (CVD) incidence [4]. Moderate-intensity cycling activity can improve cognitive performance in older adults on working memory tasks [5]. Exercise has been shown to improve the adverse changes that aging brings to the body by increasing muscle mass and reducing fat mass [15–18]. CVD accounted for over 40% of the total deaths in the population of 70-year-olds [19]. Cardiovascular disease-related disability has been seen as a disease in men for decades, but it is more common in women than in men [20,21]. J. Bellettiere et al. reported that each additional hour of daily sedentary time was associated with a 12% increase in CVD risk (HR = 1.12, 95% CI: 1.05–1.19) in their study of ethnically diverse older women (mean age 79) [22].

It is well known that exercise can improve the relevant clinical indicators of CVD. However, the metabolic changes caused by aging cannot be ignored for the elderly, and their relationship is inseparable. First, adverse changes in body composition in the elderly can cause abdominal fat release, dyslipidemia, hypertension, and increased fasting glucose, also known as metabolic syndrome (Mets) [23,24]. Dyslipidemia (high triglycerides, low high-density lipoprotein cholesterol (HDL-C), and high LDL particle levels) has been considered a significant risk factor for CVD [25,26]. Secondly, a general feature of the metabolic changes in aging is chronic inflammation. The Korea National Health and Nutrition Examination Survey from 2016 to 2017 reported that obesity was a risk factor for elevated inflammatory markers in postmenopausal women [27]. Among them, C-reactive protein (CRP) is the primary inflammatory marker, and several studies have shown that CRP has a direct proinflammatory effect and accelerates atherosclerosis [28]. Therefore, inflammation, as a risk factor, is an intrinsic mechanism for the development of cardiovascular disease [29]. Exercise may be effective in eliminating inflammation (such as lowering CRP levels in older people) to prevent and treat metabolic syndrome, according to studies [24,30–33]. Aerobic exercise is thought to be more suitable than resistance training for modulating endothelial activation and inflammatory markers in the elderly [32,34].

Due to the influence of estrogen, there are gender-specific differences in the risk of cardiovascular and cerebrovascular events. Older women often experience a greater decline in musculoskeletal health, including reduced muscle mass and strength. Additionally, older women typically have lower rates of smoking and alcohol consumption compared to men [35,36]. Given these factors, exercise emerges as one of the most accessible and universally applicable lifestyle interventions, particularly as an alternative to smoking and alcohol cessation—for promoting health in this population [37].

However, previous studies and meta-analyzes have some limitations. First, less attention has been paid to older women, especially on their cardiovascular indicators. Second, the existing meta-analyzes may have a small sample size, lack analysis of specific subgroups, and be limited by methodological heterogeneity (e.g. intervention duration). The effects of different exercise strategies on elderly women have not been sufficiently studied, and the optimal approaches and effects of specific exercise interventions to maximize benefits for older women with varying baseline physiological conditions remain unclear. The primary goal of our work was to evaluate the practical, combined benefit of structured exercise as it is commonly prescribed in practice. To this end, this systematic review and meta-analysis of randomized controlled trials (RCTs) specifically aimed to quantify the effects of supervised exercise interventions on metabolic risk, cardiovascular fitness, and body composition in healthy elderly women (aged ≥ 60 years). A key focus was to investigate the impact of intervention duration by performing subgroup analyzes. Furthermore, we addressed methodological heterogeneity through rigorous sensitivity analyzes. The ultimate goal is to provide a precise evidence base to guide the duration of tailored exercise programs for this population. This review included studies published from 2014 onward to align with key methodological advancements. This period reflects the adoption of standardized exercise reporting guidelines [38] and the establishment of device-based physical activity measurement as the gold standard [39,40]. Furthermore, this temporal scope ensures direct alignment with the evidence base underlying current clinical guidelines, guaranteeing that our findings are immediately applicable to contemporary exercise prescription frameworks [41,42].

## 2. Method

### 2.1. Data source and search strategy

**Figure 1.**
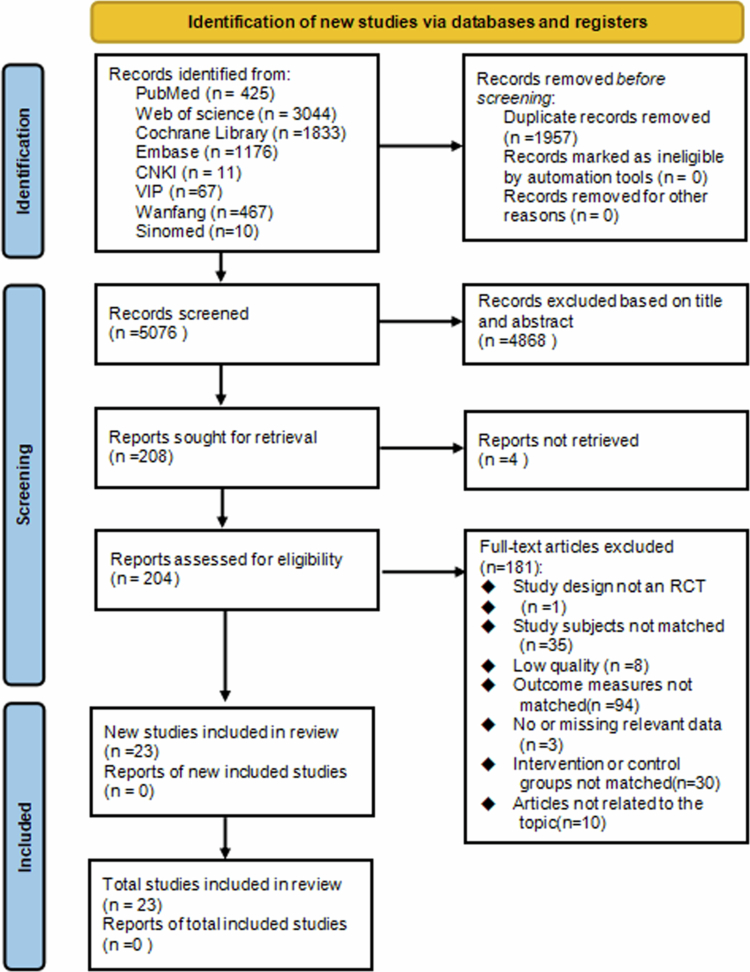
Preferred reporting Items for systematic reviews and meta-analyzes flow diagram.

The meta-analysis was performed following the Preferred Reporting Items for Systematic Reviews and Meta-Analyzes (PRISMA) guidelines (Figure 1) [43] and registered on the International Prospective Register of Systematic Reviews (PROSPERO: CRD42024599473).

Between October 2014 and October 2024, we systematically searched the following databases for eligible studies: PubMed, Cochrane Library, Web of Science, Embase, Scopus, CNKI, VIP, Wanfang, and Sinomed, with no restrictions on language or country. We also manually searched the references of published studies.

Two authors (JS and JP) independently screened the titles and abstracts of the articles through a systematic search. The search strategy included the following terms: “older women”, “ exercise,” “resistance training,” and “randomized controlled trials as a topic.” The search strategy was designed to be comprehensive and to reflect the conceptual framework of “exercise” as established in the preceding sections. Consequently, it included both the broad term “exercise” and the specific modality “resistance training” to ensure all relevant studies within our defined scope were captured. The complete search strategy is provided in Table S1. In addition, the reviews, gray literature, case reports, opinion pieces, conference abstracts, and PhD theses were excluded. In the case of disagreement, a third researcher would make a final decision.

### 2.2. Study selection

Initially, records were identified after the exclusion of duplicates. Subsequently, many records were removed during the title and abstract screening phase. Studies with missing information (such as undefined cutoff points) or ambiguous data (such as discrepancies between numbers reported in the text and tables) were excluded during the full-text screening phase. Ultimately, studies that met the following criteria were included in the quantitative meta-analysis: (1) randomized clinical trials; (2) women aged 60 years and over (the age cutoff was derived based on the United Nations definition of the old as someone beyond 60 years [44], independent for performing daily activities; (3) intervention group received supervised exercise with tolerable intensity, and volume for participants, which included aerobic or strength training; (4) control group with no exercise or low-dose activities (e.g. stretching, manual, or daily activities) with lower energy expenditure than the intervention group; (5) measurements using established methods from baseline to the final follow-up, including body composition, hemodynamic responses, and/or serum/plasma metabolic profiles. Specifically, activities for the control group were defined as light intensity (1.5–3.0 METs) [45], corresponding to ~50%–63% of maximum heart rate and a perception of “light” effort [46]. The frequency and duration of these activities were not prescribed. Still, they were expected to result in a total weekly volume substantially lower than the recommended 150 minutes of moderate-intensity exercise [42].

Studies were excluded from this systematic review and meta-analysis if they fulfilled the following criteria: (1) unhealthy populations with chronic disease at baseline (e.g. uncontrolled diabetes, uncontrolled hypertension, or undergoing hormone therapy), ineligible for study participation following cardiologist review; (2) participants with regular exercise training during the last 6 months; (3) l literature of poor quality, defined as studies with a high risk of bias (as determined by the Cochrane RoB2 tool), critical missing data necessary for quantitative synthesis that could not be obtained, or evidence of selective outcome reporting, non-Chinese/English literature, and high-quality literature that uses the same data.

### 2.3. Data extraction and quality assessments of each study

Two reviewers (JS and JP) screened full texts for suitability according to the inclusion and exclusion criteria using Covidence [47]. Data extraction was also independently conducted by the same researchers. The following data were extracted from each included study and entered into a custom-built spreadsheet: general study information (author, year of publication, country or area in which the data were collected in Table 1), participants' characteristics (number of patients included, mean population age, data on baseline participant traits), intervention components (exercise frequency, intensity, time), and postintervention results, including body composition, metabolic profile, and cardiovascular fitness. For studies with multiple intervention arms, data from each intervention group versus the control group (CG) were extracted simultaneously. The extracted data were finally verified for accuracy by a third researcher. Detailed intervention parameters, including intensity, supervision, adherence, and prescription details, are provided in Supplementary Table S2. For monitoring adherence in the exercise groups, attendance was systematically recorded at each supervised session across all included studies. Adherence was calculated as the percentage of completed sessions out of the total prescribed.

**Table 1.** Characteristics of the 23 included studies.

Author, year, country/region	Intervention		Sample size(*n*)		Age(yeas)		Frequency	Treatment duration	Total intervention period	outcomes
	E group	C group	E group	C group	E group	C group				
P. M. Cunha [48], Brazil	8 exercises: chest press, horizontal leg press, seated row, knee extension, preacher curl (free weights), leg curl, triceps pushdown, and seated calf raise	No structured exercise program	*P*. M. Cunha, 2021a: 19 *P*. M. Cunha, 2021b: 18	18	*P*. M. Cunha, 2021a: 70.3 ± 6.3 *P*. M. Cunha, 2021b: 68.7 ± 4.7	69.0 ± 4.2	*P*. M. Cunha, 2021a: 3 times/week, 15–20 minutes/per time *P*. M. Cunha, 2021b: 3 times/week, 45–60 minutes/per time	12w	16w	TG, TC, HDL-c, LDL-c, GLU, CRP, TF, RF
C. M. Tomeleri [49], Brazil	8 exercises: chest press, seated row, triceps pushdown, preacher curl, horizontal leg press, knee extension, knee curl, and seated calf raise	No physical exercise	24	22	71.0 ± 5.4	68.8 ± 4.6	3 times/week, 45–60 minutes/per time	12w	16w	CRP, RF, SMM
C. Gómez-Tomás [50], Spain	6 exercises: progressive load and intensity with elastic resistance band.	No training	18	20	70.89 ± 4.42	70.45 ± 5.44	3 times/week, 50 minutes/per time	1year	1year	TG, TC, HDL-c, LDL-c, CRP, Weight, WC
R. R. Porter [26], United States	Moderate-dose group: 14 kcal/kg/week exercise	Low-dose group: 8 kcal/kg/week exercise	30	35	64.6 ± 3.6	65.6 ± 4.7	E:3-4 times/week, 163.5 ± 12.6 minutes/per time C:3-4 times/week, 108.5 ± 9.1 minutes/per time	16w	16w	VO2max, TG, HDL-c, Weight
H. C. M. de Souza [51], Brazil	H. C. M. de Souza, 2024a: Sham inspiratory muscle training associated with whole-body vibration H. C. M. de Souza, 2024b: Sham inspiratory muscle training associated with sham whole-body vibration	Inspiratory muscle training associated with whole-body vibration	H. C. M. de Souza, 2024a: 14 H. C. M. de Souza, 2024b: 14	14	H. C. M. de-Souza, 2024a: 68.78 ± 4.56 H. C. M. de Souza, 2024b: 68.71 ± 4.27	67.71 ± 3.95	The WBV time in the first two weeks of intervention was 10 minutes, increasing to 15 minutes in the 3rd week, 20 minutes in the 5th week, and 30 minutes in the 5th week of training. Inspiratory muscle training was performed with a weekly frequency of 7 days and a series of 60 repetitions daily.	12 W	12 W	TF, RF, FFM
M. D. M. Stojanović [52], Serbia	12 chair-based exercises: knee, hip, shoulder, elbow, trunk extension and flexion, hip abduction and adduction with elastic band	Institution-based activities: chess, dice, reading, and crafts	86	82	75.7 ± 8.9	74.5 ± 8.2	2 times/week, 55–60 minutes/per time	12w	12w	TG, TC, LDL, GLU
J. Rodrigues-Krause [53], Brazil	J. Rodrigues-Krause, 2018a: A dance-based intervention incorporating elements from various styles to enhance balance, flexibility, muscle power, and aerobic conditioning, structured into five parts: warm-up, across-the-floor, choreography, show, and cool-down, with intensity guided by song BPM J. Rodrigues-Krause, 2018b: Walking sessions with dynamic joint mobilization, static stretching, treadmill walking at varying intensities, and cool-down stretching.	Stretching exercises for large muscle groups performed standing or seated, focusing on gentle movements, postural alignment, and breath control without external load or music	J. Rodrigues-Krause, 2018a: 10 J. Rodrigues-Krause, 2018b: 10	10	J. Rodrigues-Krause, 2018a: 66 ± 5.65 J. Rodrigues-Krause, 2018b: 64 ± 2.42	66 ± 7.26	E:3 times/week, 60 minutes/per time C:1 times/week, 50 minutes/per time	8 W	8 W	VO2max, TG, TC, HDL-c, LDL-c, GLU, CRP, insulin, weight, WC
C. M. Tomeleri [54], Brazil	C. M. Tomeleri, 2023a: 8 exercises: chest press, seated row, triceps pushdown, preacher curl, horizontal leg press, leg extension, leg curl, and seated calf raise C. M. Tomeleri, 2023b: 8 exercises: preacher curl, triceps pushdown, seated row, chest press, seated calf raise, leg curl, leg extension, and horizontal leg press	No training	C. M. Tomeleri, 2023a: 15 C. M. Tomeleri, 2023b: 14	15	≧60	≧60	3 times/week, the RT sessions consisted of 3 sets of 10–15 repetitions per exercise	12 W	16 W	TG, TC, HDL-c, LDL-c, GLU, CRP, RF
L. Macêdo Santiago [55], Brazil	8 exercises in bi-set method for lower and upper limbs: leg press, supine, knee extension, pulley (back), knee flexion, elbow flexion, leg press, and elbow extension	No intervention	19	10	63.0 ± 2.0	63.0 ± 1.0	3 times/week, 50 minutes/per time	8w	8w	RF, Weight
H. M. Elsangedy [56], Brazil	8 exercises: bench press, leg press, lateral pulldown, knee extension, lateral shoulder raise, knee curl, biceps curl, triceps pushdown	Board games and manual activities	16	16	65.7 ± 3.3	66.3 ± 2.8	3 times/week, the RT sessions consisted of 3 sets of 15 repetitions per exercise	12w	18w	VO2max, RF, Weight, FFM
J. Kortas [57], Poland	Three microcycles of Nordic Walking (NW) training: initial functional efficiency, endurance improvement, and final intensity increase	Physical activity	18	18	66.78 ± 4.76	66.12 ± 4.83	3 times/week, 65–75 minutes/per time	12w	12w	GLU, Insulin
W. H. Son [58], Korea	Walking exercise with 20-min warm-up, intensity at 64%–76% HRmax	No exercise regularly	14	12	70.2 ± 1.21	69.9 ± 1.14	100 steps/min	12w	12w	RF, Weight, SMM
T. C. M. [59], Brazil	8 exercises: chest press, seated row, triceps pushdown, preacher curl, horizontal leg press, knee extension, knee curl and seated calf raise	No physical exercise	22	23	72.1 ± 6.3	68.8 ± 4.9	3 times/week, the RT sessions consisted of 3 sets of 10–15 repetitions per exercise	12w	18w	SBP, DBP, TG, HDL-c, GLU, CRP, RF, WC, SMM
V. Teixeira do Amaral [60], Brazil	V. Teixeira do Amaral, 2024a: High-intensity interval training combined with resistance training in three clusters V. Teixeira do Amaral, 2024b: Moderate intensity continuous train- ing combined with RT in two clusters	Community-based exercise programs for promoting regular exercise in groups with similar conditions	V. Teixeira do Amaral, 2024a: 34 V. Teixeira do Amaral, 2024b: 38	20	V. Teixeira do Amaral, 2024a: 75 ± 6 V. Teixeira do Amaral, 2024b: 74 ± 6	73 ± 6	2 times/week, 30–60 minutes/per time	9m	12m	Weight, WC
R. R. Costa [61], Brazi	R. R. Costa, 2019a: Interval training for water-based aerobic training (WA) group: 90–100% HR for stimulus periods and 80%–90% HR for recovery, with increasing intensity R. R. Costa, 2019b: Water-based resistance training (WR) group: 80-second exercises at maximum speed, with 4 sets of 20 seconds in the first 5 weeks and 8 sets of 10 seconds in the last 5 weeks, and active recovery intervals	Nonperiodized relaxation exercises in immersion	R. R. Costa, 2019a: 23 R. R. Costa, 2019b: 23	23	R. R. Costa, 2019a: 66.80 (64.55 to 69.05) R. R. Costa, 2019b: 66.78 (64.41 to 69.15)	64.63 (62.23 to 67.03)	2 times/week, 45 minutes/per time	10w	10w	TG, TC, LDL
R. R. Costa [62], Brazi	R. R. Costa, 2018a: water-based aerobic training group: Bilateral performance combining upper and lower limbs, with warm-up and stretching R. R. Costa, 2018b: Water-based resistance training group: upper limb exercises bilaterally, lower limb exercises unilaterally, performed at maximal effort and amplitude for maximum velocity and resistance	Nonperiodic water-based exercise program with stretching, relaxation, and coordination games	R. R. Costa, 2018a: 23 R. R. Costa, 2018b: 23	23	R. R. Costa, 2018a: 66.80 (64.55 to 69.05) R. R. Costa, 2018b: 66.78 (64.41 to 69.15)	64.63 (62.23 to 67.03)	2 times/week, 45 minutes/per time	10w	10w	VO2max
L. S. Andrade [63], Brazil	Each training session: stationary running, frontal kick, and cross-country skiing, with intensity progressively increased based on Borg's RPE 6-20 Scale.	A 1:1 effort-to-rest ratio compared to E Group	16	16	64.8 ± 3.6	64.8 ± 3.6	2 times/week, 44 minutes/per time	12w	12w	HRmax, VO2max
M. S. Häfele [64], Brazil	M. S. Häfele, 2023a: Aquatic training with intensity based on HR at anaerobic threshold, using exercises like stationary running, frontal kick, cross-country skiing, and butt kicks, with continuous and interval training phases M. S. Häfele, 2023b: Aquatic training combined with resistance training using two blocks of upper and lower limb exercises, performed at maximal effort with increasing duration and intervals, and integrated with continuous training	water-based therapeutic sessions with slow, self-performed exercises targeting mobility, breathing, relaxation, massage, and stretching to avoid significant neuromuscular and cardiorespiratory adaptations	M. S. Häfele, 2023a: 17 M. S. Häfele, 2023b: 18	17	M. S. Häfele, 2023a: 67.06 ± 4.58 M. S. Häfele, 2023b: 66.00 ± 3.77	65.41 ± 3.66	E:2 times/week, 45 minutes/per time C:1 times/week, 30 minutes/per time	16w	16w	HRmax, SBP, DBP
M. Carrasco-Poyatos [65], Spain	Warm-up with dynamic range of motion, Pilates main or muscular training session, and cool-down with static range of motion and breathing exercises	Normal physical activity habits	M. Carrasco-Poyatos, 2019a: 16 M. Carrasco-Poyatos, 2019b: 19	12	M. Carrasco-Poyatos, 2019a: 67.5 ± 3.87 M. Carrasco-Poyatos, 2019b: 73.36 ± 4.84	65.89 ± 4.54	2 times/week, 60 minutes/per time	18w	18w	FFM
F. Urzi [66], Slovenia	8 exercises: chair squats, biceps curl, seated row, knee extension, leg press, hip abduction, knee flexion, calf rise	No any placebo or treatment.	11	9	84.4 (7.7)	88.9 (5.3)	3 times/week, Each ERT session consisted of a general warmup of 10 minutes and 35 to 40 minutes of 8 resistance exercises	12w	12w	CRP, Glu,
A. M. Monteiro [67], Portugal	A. M. Monteiro, 2022a: Warm-up, aerobic exercises, resistance training with elastic bands and free weights, balance training with sticks, balls, and balloons, and cool-down with breathing and stretching A. M. Monteiro, 2022b: Warm-up, resistance training with elastic bands and free weights, aerobic exercises, balance training with sticks, balls, and balloons, and cool-down with breathing and stretching	No physical exercise program	A. M. Monteiro, 2022a: 30 A. M. Monteiro, 2022b: 32	29	A. M. Monteiro, 2022a: 69.40 ± 5.24 A. M. Monteiro, 2022b: 70.63 ± 5.15	68.72 ± 5.09	3 times/week, 60 minutes/per time	16w	16w	FFM
S. L. Oh [68], South Korea	Elastic band resistance training program with supervised and self-directed phases, focusing on upper and lower body exercises, progressing intensity every 4 weeks	Continue routine daily activities with weekly supervised stretching	19	19	74.9 ± 1.5	73.5 ± 1.2	2 times/week, 60 minutes/per time	18w	18w	Weight, FFM
H. Blain [69], France	Moderate-intensity walking training in a city park, gradually increasing heart rate target from 40% to 60%–80% of maximal heart rate (calculated using Tanaka's equation)	Physical activity	51	47	65.58 (4.44)	65.78 (4.15)	3 times/week, 50 minutes/per time	6m	6m	Weight

Abbreviations: C group = Control group; CRP = C-reactive protein; DBP = Diastolic blood pressure; E group = Experimental group; FFM = Fat-free mass; Glu = Glucose; HDL-C = High-density lipoprotein cholesterol; HRmax = Maximal heart rate; LDL = Low-density lipoprotein; LDL-C = Low-density lipoprotein cholesterol; MD = Mean Difference; RF = Relative body fat; SBP = Systolic blood pressure; SMM = Skeletal muscle mass; TC = Total cholesterol; TFM = Total fat mass; TG = Triglycerides; VO₂peak = Maximal oxygen uptake; WC = Waist circumference.

The RCTs were evaluated for risk of bias and overall quality using the Revised Cochrane Risk of Bias Tool for Randomized Trials (RoB2) [70]. The RoB2 tool evaluates five domains, which include (1) bias arising from the randomization process; (2) bias due to deviations from intended interventions; (3) bias due to missing outcome data; (4) bias in the measurement of the outcome; and (5) bias in the selection of the reported result. After evaluating these domains, the overall bias was assessed using low, medium, and high ratings. The overall risk of bias for each study was assessed using the Cochrane RoB 2.0 tool. Studies were rated as “low risk” only if all domains were low risk; “high risk” if any domain was high risk or if multiple domains raised some concerns that substantially undermined confidence; and “some concerns” otherwise.

### 2.4. Certainty of the evidence: GRADE approach

In the GRADE system, the certainty of evidence from randomized controlled trials (RCTs) is typically categorized into four levels: high, moderate, low, and very low [71]. RCTs are initially assigned a high certainty of evidence, but this can be downgraded based on the following factors: (1) risk of bias: serious limitations in study design or execution (assessed by RoB2); (2) inconsistency: unexplained heterogeneity in results (I² > 50% or poor overlap of confidence intervals); (3) indirectness: evidence indirect to the population, intervention, or outcome of interest; (4) imprecision: wide confidence intervals or small sample size (<400 participants in each study arm); and (5) publication bias: significant evidence of small-study effects. The certainty was rated as high, moderate, low, or very low. Divergences in assessments were resolved through discussion among the reviewers.

### 2.5. Data syntheses and analyzes

Two reviewers (JS and JP) extracted data in duplicate and cross-checked the results. All outcome measures were extracted and analyzed, including baseline and postintervention means ± standard deviations, and the mean difference (MD) with a 95% confidence interval was reported. If not reported, the MD between pre- and postintervention was calculated by subtracting the baseline values from the postintervention values. A correlation coefficient of 0.5 was used for outcomes for which the correlation coefficient could not be calculated. In one study where post-SD was unavailable, we used pre-SD and post-SD to estimate the SD of the change value [72]. Continuous outcomes were extracted into an electronic database as a baseline and postintervention MD ± standard deviation (SD).

Meta-analysis was conducted using the Review Manager Software. The MD was used as the summary statistic because all the studies included in this systematic review could be harmonized through unit conversion to assess the same outcome using the same measurement units (e.g. weight change measured in kilograms). Data from intention-to-treat analyzes were entered whenever available in the included RCTs. Weighted proportions were calculated based on the sample sizes of the individual studies. A *P*-value of less than 0.05 in a Z-test analysis indicated a statistically significant difference in the effect size from zero. When data were available, the pooled effect was calculated using the fixed-effect model, and no significant heterogeneity was detected. Otherwise, the random-effect model was applied. We explored heterogeneity between studies using a strategy, and we performed sensitivity analyzes (Table S3) to improve the robustness of our findings. We conducted sensitivity analyzes using a leave-one-out approach. This approach involves removing one study at a time to assess the effect on the overall outcome and the effect of individual studies on the collective outcome. We conducted subgroup analyzes to explore differences in study outcomes based on intervention duration and to further understand the sources of heterogeneity in effect sizes or underlying factors (S3).

### 2.6. Publication bias

The assessment suite included visual inspection of funnel plots, Begg's and Egger's regression test for robustness. We acknowledge that the statistical power of these tests is limited when the number of studies is small (e.g. <10) [73]. Given that the number of included studies for each outcome was small (all < 10), quantitative methods such as funnel plots and Egger's test are statistically underpowered and yield unreliable conclusions in this context. Therefore, these quantitative tests were not performed for any outcome in the present study. Trim-and-fill analysis was not performed due to methodological limitations in small meta-analyzes, as simulation studies have shown it may produce unreliable estimates when the number of studies is limited [74,75].

As an alternative, a systematic search of gray literature was conducted, including queries of clinical trial registries (e.g. ClinicalTrials.gov, WHO International Clinical Trials Registry Platform (ICTRP)) and relevant conference abstracts (International Society of Sports Nutrition (ISSN) Annual Conference, American College of Sports Medicine (ACSM)), to identify potentially omitted unpublished studies. The qualitative review did not identify a substantial body of completed but unpublished randomized controlled trials with findings contrary to the direction of the pooled results in this meta‑analysis.

## 3. Results

### 3.1. Search process

The PRISMA flow diagram (Figure 1) shows that 7033 full-text articles were identified through the screening process. After excluding duplicates, articles without accessible full texts, and those deemed irrelevant based on titles and abstracts, 204 studies were assessed for eligibility according to the PICOS criteria. Subsequently, 181 studies were excluded for not meeting the inclusion criteria. Therefore, 23 RCTs (RT groups (*n* = 33) and CG (*n* = 23)) were included in the quantitative synthesis.

### 3.2. Characteristics of the included studies

Twenty-three published between 2014 and 2024 were included, all incorporating quantitative analysis: 23 intervention RCTs groups with body composition data, 14 with serum/plasma metabolic data, and 10 with hemodynamic response data.

**Figure 2.**
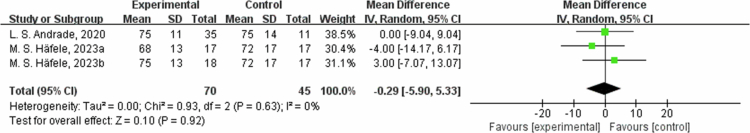
Forest plot of maximal heart rate (HRmax). Means, standard deviations (SD), and 95% confidence intervals (CI) are presented in bpm.

**Figure 3.**
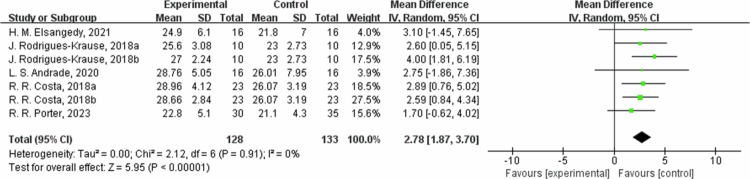
Forest plot of maximal oxygen uptake (VO2peak). Means, standard deviations (SD), and 95% confidence intervals (CI) are presented in mL/kg/min.

**Figure 4.**
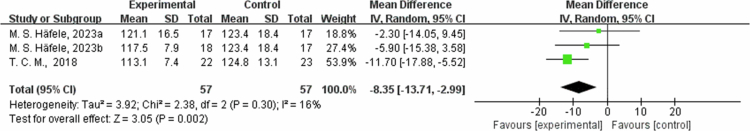
Forest plot of systolic blood pressure (SBP). Means, standard deviations (SD), and 95% confidence intervals (CI) are presented in mmHg.

**Figure 5.**
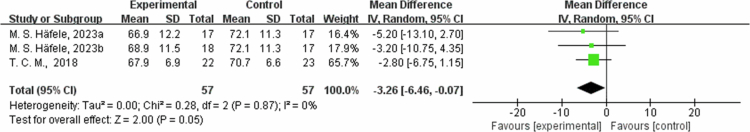
Forest plot of diastolic blood pressure (DBP). Means, standard deviations (SD), and 95% confidence intervals (CI) are presented in mmHg.

**Figure 6.**
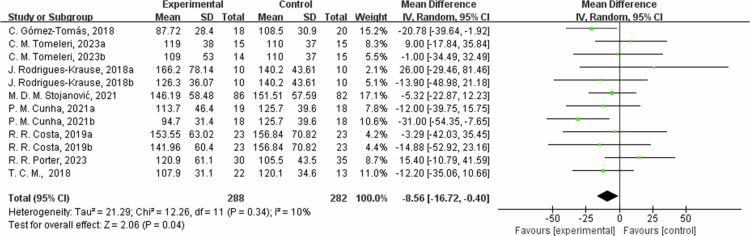
Forest plot of triglycerides (TG). Means, standard deviations (SD), and 95% confidence intervals (CI) are presented in mg/dL.

**Figure 7.**
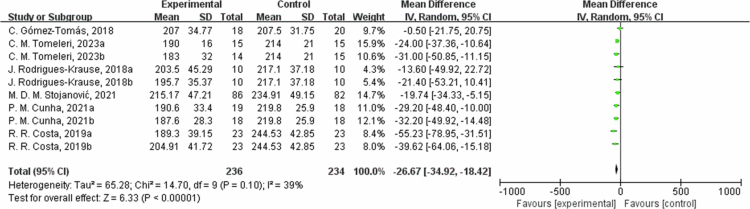
Forest plot of total cholesterol (TC). Means, standard deviations (SD), and 95% confidence intervals (CI) are presented in mg/dL.

**Figure 8.**
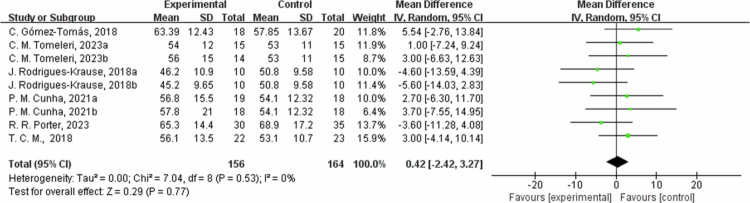
Forest plot of high-density lipoprotein cholesterol (HDL-C). Means, standard deviations (SD), and 95% confidence intervals (CI) are presented in mg/dL.

**Figure 9.**
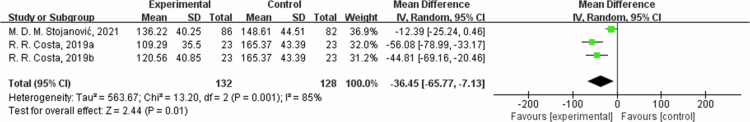
Forest plot of low-density lipoprotein cholesterol (LDL-C). Means, standard deviations (SD), and 95% confidence intervals (CI) are presented in mg/dL.

**Figure 10.**
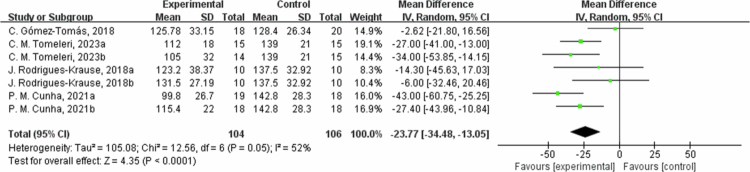
Forest plot of LDL-C (low-density lipoprotein cholesterol). Means, standard deviations (SD), and 95% confidence intervals (CI) are presented in mg/dL.

**Figure 11.**
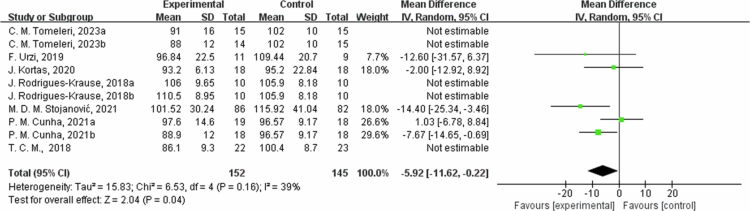
Forest plot of glucose (Glu). Means, standard deviations (SD), and 95% confidence intervals (CI) are presented in mg/dL.

**Figure 12.**
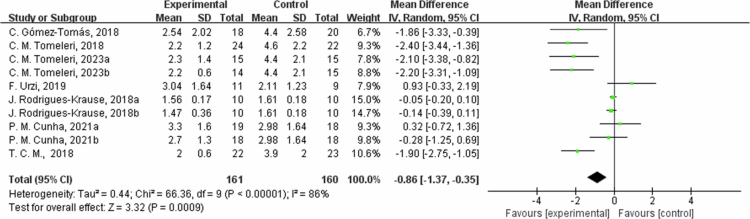
Forest plot of C-reactive protein (CRP). Means, standard deviations (SD), and 95% confidence intervals (CI) are presented in mg/L.

**Figure 13.**
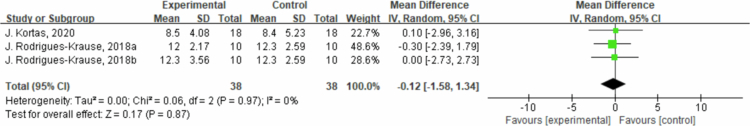
Forest plot of Insulin. Means, standard deviations (SD), and 95% confidence intervals (CI) are presented in μU/mL.

**Figure 14.**
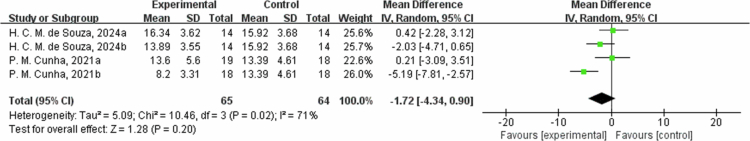
Forest plot of trunk fat mass (TFM). Means, standard deviations (SD), and 95% confidence intervals (CI) are presented in kg.

**Figure 15.**
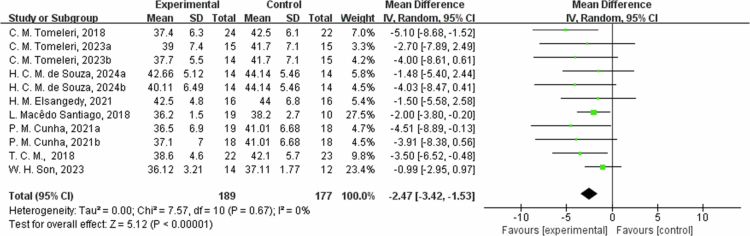
Forest plot of relative body fat (RF). Means, standard deviations (SD), and 95% confidence intervals (CI) are presented in %.

**Figure 16.**
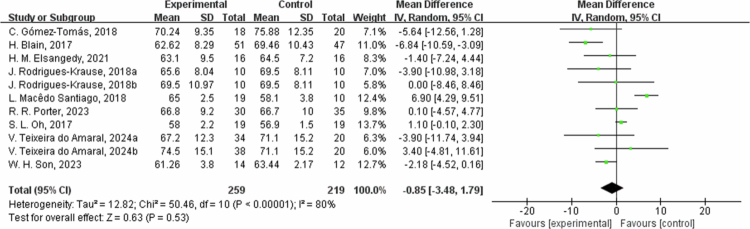
Forest plot of weight. Means, standard deviations (SD), and 95% confidence intervals (CI) are presented in kg.

**Figure 17.**
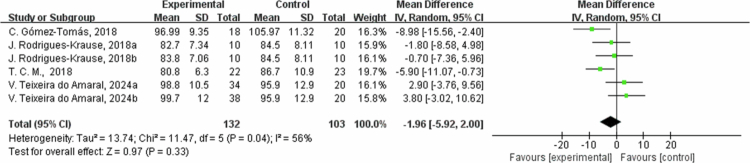
Forest plot of waist circumference (WC). Means, standard deviations (SD), and 95% confidence intervals (CI) are presented in cm.

**Figure 18.**
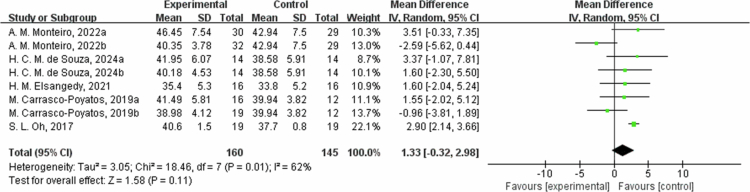
Forest plot of fat-free mass (FFM). Means, standard deviations (SD), and 95% confidence intervals (CI) are presented in kg.

**Figure 19.**
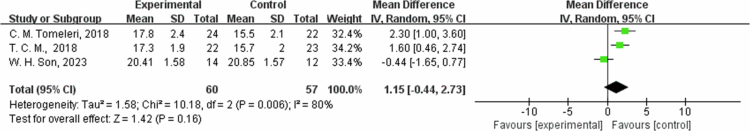
Forest plot of skeletal muscle mass (SMM). Means, standard deviations (SD), and 95% confidence intervals (CI) are presented in kg.

Characteristics of the interventions and comparators. The study population was recruited through advertisements or screened for eligibility at universities or research institutions. The study populations from Brazil accounted for 648 individuals (53.4%) across 15 publications, while those from South Korea accounted for 64 individuals (5.3%) across 2 publications. The remaining participants were from European and American countries. This review includes various forms of exercise, including water-based activities, resistance band exercises, brisk walking, and dancing. The characteristics of the interventions and comparators included in the systematic review with meta-analysis are presented in Table 1. All results and subgroup analyzes are presented in forest plots in Figures 2–19 and Figure S1.

Additionally, all studies included in the quantitative synthesis were found to have some overall risk of bias across all outcomes. The quality of this meta-analysis's overall quantitative outcome was determined using the GRADE tool, which objectively assesses outcomes based on different domains using a scoring system (Table S4). Evidence was moderate for improvements in triglycerides (TG), relative body fat (RF), defined as body fat percentage, total cholesterol (TC), maximal oxygen uptake (VO2peak), and systolic blood pressure (SBP). The evidence for the remaining outcomes was rated as low certainty.

### 3.3. Effects of exercise on cardiovascular fitness

The effects of exercise on metabolic risk included in the systematic review with meta-analysis are presented in Table 2. In terms of VO2peak, data from five studies indicate that the VO2peak of the intervention group increased by an average of 2.78 ml/kg/min 95% CI: 1.87, 3.70; I² = 0%, *P* < 0.00001). A sensitivity analysis was not performed due to the low heterogeneity. To further verify the stability of the results, a subgroup analysis was frequently performed according to the intervention (Table S5). Subgroup analysis based on intervention duration showed that in one trial with an intervention duration of ≤8 weeks, VO2peak increased by 3.41 ml/kg/min (95% CI: 1.75, 5.07, *P* < 0.0001, I² = 0%); in the three trials with 8 to 12 weeks, VO2peak increased by 2.74 ml/kg/min (95% CI: 1.50, 3.99, *P* < 0.0001, I² = 0%); and in one trial with >12 weeks, VO2peak increased by 1.70 ml/kg/min (95% CI: −0.62, 4.02, *P* = 0.15). For maximal heart rate (HRmax), the results of two studies show no significant difference between the intervention group and the control group (MD = −0.29, 95% CI: −5.90, 5.33, *P* = 0.92). A sensitivity analysis was not performed due to the low heterogeneity. Subgroup analysis by intervention duration showed that in one trial with 8–12 weeks, HRmax changed by 0.00 bpm (95% CI: −9.04, 9.04, *P* = 1); and in one trial with intervention duration >12 weeks, HRmax changed by −0.46 bpm (95% CI: −7.62, 6.69, *P* = 0.90). SBP was reduced by an average of 8.35 mmHg (95% CI: −13.71, −2.99, *P* = 0.002), and diastolic blood pressure (DBP) was reduced by an average of 3.26 mmHg (95% CI: −6.46, −0.07, *P* = 0.05). For SBP outcome measures, heterogeneity I^2^ was 16% with *P*-value = 0.002. In the sensitivity analysis, removing M. S. Hafele 2023a [64] study, heterogeneity I^2^ was 16%, and the source of heterogeneity may be the difference in sample content and interventions. Subgroup analysis by intervention duration showed that in one trial lasting 8 to 12 weeks, SBP decreased by −11.70 mmHg (95% CI: −17.88, −5.52, *P* = 0.0002); and in one trial lasting >12 weeks, SBP decreased by −4.48 mmHg (95% CI: −11.86, 2.90, *P* = 0.23). For DBP outcome measures, heterogeneity I² was 0% with *P*-value = 0.05. A sensitivity analysis was not performed due to the low heterogeneity. Subgroup analysis by intervention duration showed that in one trial with 8 to 12 weeks, DBP decreased by −2.80 mmHg (95% CI: −6.75, 1.15, *P* = 0.16); and in one trial with intervention duration >12 weeks, DBP decreased by −4.15 mmHg (95% CI: −9.62, 1.31, *P* = 0.14).

**Table 2.** Summary of results.

Outcome	Trials	Participant	Statistical method	Effect estimate	Heterogeneity(I2)	*P-*value
HRmax	2	98	Mean Difference (IV, Random, 95% CI)	−0.29 [−5.90, 5.33]	0%	0.92
VO2peak	5	228	Mean Difference (IV, Random, 95% CI)	2.78 [1.87, 3.70]	0%	<0.00001
SBP	2	97	Mean Difference (IV, Random, 95% CI)	−8.35 [−13.71, −2.99]	16%	0.002
DBP	2	97	Mean Difference (IV, Random, 95% CI)	−3.26 [−6.46, −0.07]	0%	0.05
TG	8	504	Mean Difference (IV, Random, 95% CI)	−8.56 [−16.72, −0.40]	10%	0.04
TC	6	404	Mean Difference (IV, Random, 95% CI)	−26.67 [−34.92, −18.42]	39%	<0.00001
HDL-C	6	277	Mean Difference (IV, Random, 95% CI)	0.42 [−2.42, 3.27]	0%	0.77
LDL	2	237	Mean Difference (IV, Random, 95% CI)	−36.45 [−65.77, −7.13]	85%	0.01
LDL-C	4	167	Mean Difference (IV, Random, 95% CI)	−23.77 [−34.48, −13.05]	52%	<0.0001
Glu	7	398	Mean Difference (IV, Random, 95% CI)	−6.67 [−11.59, −1.75]	70%	0.008
CRP	7	278	Mean Difference (IV, Random, 95% CI)	−0.86 [−1.37, −0.35]	86%	0.0009
Insulin	2	66	Mean Difference (IV, Random, 95% CI)	−0.12 [−1.58, 1.34]	0%	0.87
TFM	2	97	Mean Difference (IV, Random, 95% CI)	−1.72 [−4.34, 0.90]	71%	0.20
RF	8	319	Mean Difference (IV, Random, 95% CI)	−2.47 [−3.42, −1.53]	0%	<0.00001
Weight	9	448	Mean Difference (IV, Random, 95% CI)	−0.85 [−3.48, 1.79]	80%	0.53
WC	4	205	Mean Difference (IV, Random, 95% CI)	−1.96 [−5.92, 2.00]	56%	0.33
FFM	5	250	Mean Difference (IV, Random, 95% CI)	1.33 [−0.32, 2.98]	62%	0.11
SSM	3	117	Mean Difference (IV, Random, 95% CI)	1.15 [−0.44, 2.73]	80%	0.16

Abbreviations: CRP = C-reactive protein; DBP = Diastolic blood pressure; FFM = Fat-free mass; Glu = Glucose; HDL-C = High-density lipoprotein cholesterol; HRmax = Maximal heart rate; LDL = Low-density lipoprotein; LDL-C = Low-density lipoprotein cholesterol; MD = Mean Difference; RF = Relative body fat; SBP = Systolic blood pressure; SMM = Skeletal muscle mass; TC = Total cholesterol; TFM = Total fat mass; TG = Triglycerides; VO₂peak = Maximal oxygen uptake; WC = Waist circumference.

### 3.4. Effects of exercise on metabolic risk

The effects of exercise on metabolic risk included in the systematic review with meta-analysis are presented in Table 2. The results from eight studies showed that the intervention group had an average reduction of 8.56 mg/dL in TG levels (95% CI: −16.72, −0.40, *P* = 0.04). For TG outcome measures, the heterogeneity was low (I^2^ = 10%), excluding R. R. Porter 2023 [26] study, the effect size increased and more significant (MD = −11.03,95% CI: −18.97, −3.10, *P* = 0.006), possibly due to different intervention intensity in the control group, Subgroup analysis by intervention duration showed that in one trial with ≤8 weeks, TG changed by 0.03 mg/dL (95% CI: −37.25, 37.31, *P* = 1, I² = 30%); in five trials with 8 to 12 weeks, TG decreased by −9.55 mg/dL (95% CI: −18.71, −0.40, *P* = 0.04, I² = 0%); and in two trials with >12 weeks, TG decreased by −3.88 mg/dL (95% CI: −39.26, 31.50, *P* = 0.83, I² = 79%).

Regarding TC, the results from six studies showed that the intervention group had an average reduction of 26.67 mg/dL in TC (95% CI: −34.92, −18.42, *P* < 0.00001). The heterogeneity was moderate for the TC outcome measures (I^2^ = 39%). In the sensitivity analysis, excluding C. Gomez-Tomas, 2018 [50] and R. R. Costa, 2019a [61] study, the effect size changed slightly but remained significant (MD = −26.42, 95% CI: −33.14, −19.70, *P* < 0.00001). The differences in training style, intensity, frequency, and duration between the two interventions studied may have contributed to the heterogeneity of the TC. In the subgroup analysis by intervention duration, in one trial with ≤8 weeks, TC decreased by −18.01 mg/dL (95% CI: −41.94, 5.91, *P* = 0.14, I² = 0%); in four trials with 8 to 12 weeks, TC decreased by −30.13 mg/dL (95% CI: −37.86, −22.40, *P* < 0.00001, I² = 22%); and in one trial with >12 weeks, TC changed by -0.50 mg/dL (95% CI: −21.75, 20.75, *P* = 0.96).

However, for HDL-C, the results from six studies showed no significant difference between the intervention group and the control group (MD = 0.42 mg/dL, 95% CI: −2.42, 3.27, *P* = 0.77). The sensitivity analysis found no significant changes for the low HDL-C heterogeneity (I^2^ = 0%). Subgroup analysis by intervention duration showed that in one trial with ≤8 weeks, HDL-C changed by −5.13 mg/dL (95% CI: −11.28, 1.02, *P* = 0.10, I² = 0%); in three trials with 8 to 12 weeks, HDL-C changed by 2.58 mg/dL (95% CI: −1.33, 6.49, *P* = 0.20, I² = 0%); and in two trials with >12 weeks, HDL-C changed by 0.83 mg/dL (95% CI: −8.12, 9.78, *P* = 0.86, I² = 60%). For LDL-C (low-density lipoprotein cholesterol), the results from four studies showed that the intervention group had an average reduction of 23.77 mg/dL in LDL-C levels (95% CI: −34.48, −13.05, *P* < 0.0001). The heterogeneity was moderate for the LDL-C outcome measures (I^2^ = 52%). After excluding C. Gomez-Tomas, 2018 [50] and *P*. M. Cunha, 2021a [48] study, the effect size increased and became more significant (MD = −25.28, 95% CI: −33.81, −16.74, *P* < 0.00001), which may be mainly due to differences in study subjects and interventions. In the subgroup analysis by intervention duration, in one trial with ≤8 weeks, LDL-C changed by −9.46 mg/dL (95% CI: −29.67, 10.76, *P* = 0.36, I² = 0%); in two trials with 8 to 12 weeks, LDL-C decreased by −31.84 mg/dL (95% CI: −40.16, −23.53, *P* < 0.00001, I² = 0%); and in one trial with >12 weeks, LDL-C changed by −2.62 mg/dL (95% CI: -21.80, 16.56, *P* = 0.79).

In addition, the intervention measures also showed significant reductions in blood glucose(Glu) and CRP, with average reductions of 5.59 mg/dL (95% CI: −10.12, −1.06, *P* = 0.02) and 0.86 mg/L (95% CI: −1.37, −0.35, *P* = 0.0009), respectively. For Glu outcome measures, high heterogeneity (I^2^ = 70%), In the sensitivity analysis, excluding C. M. Tomeleri, 2023a [54], C. M. Tomeleri, 2023b [54]; J. Rodrigues-Krause, 2018a [53]; J. Rodrigues-Krause, 2018b [53] and T. C. M., 2018 [59] study, the effect size decreased but still significant (MD = −5.54, 95% CI: −9.75, −1.33, *P* = 0.01). It may be related to factors such as interventions and sample characteristics of different studies. Subgroup analysis by intervention duration showed that in one trial with ≤8 weeks, Glu changed by 2.45 mg/dL (95% CI: −2.98, 7.87, *P* = 0.38, I² = 0%); and in six trials with 8 to 12 weeks, Glu decreased by −9.29 mg/dL (95% CI: −13.73, −4.85, *P* < 0.0001, I² = 51%). High heterogeneity for CRP outcome measures (I^2^ = 86%). In the sensitivity analysis, excluding T. C. M., 2018 [59] and C. M. Tomeleri, 2018 [49] study, the effect size decreased but remained significant (MD = −0.47,95% CI: −0.92, −0.03, *P* = 0.04), which may be sources of heterogeneity due to differences in study design, interventions, and measures of outcome measures. Subgroup analysis by intervention duration showed that in one trial with ≤8 weeks, CRP changed by −0.07 mg/L (95% CI: −0.21, 0.06, *P* = 0.26, I² = 0%); in five trials with 8 to 12 weeks, CRP decreased by −1.10 mg/L (95% CI: −2.07, −0.13, *P* = 0.03, I² = 83%); and in one trial with >12 weeks, CRP decreased by −1.86 mg/L (95% CI: −3.33, −0.39, *P* = 0.01). The summary results of two studies on low-density lipoprotein (LDL) showed that the average reduction in LDL in the intervention group was 36.45 mg/dL (95% CI: −65.77, −7.13), with heterogeneity of I² = 85% and a *P*-value of 0.01, it indicates that the intervention measures have a significant effect on reducing LDL levels. Heterogeneity I^2^ was 85% for the LDL indicator with a *P* value = 0.01. Due to the high heterogeneity, a sensitivity analysis was performed, identifying M. D. M. Stojanovic, 2021 [52] study as a possible source of heterogeneity. After excluding this study, the effect was estimated at −50.79 mg/dL (95% CI: −67.48, −34.10, *P* < 0.00001), and I^2^ was 0%. The source of heterogeneity may be methodological differences in sample characteristics, interventions, and outcome measures determination. For subgroup analysis by intervention duration, in two trials with 8 to 12 weeks, LDL decreased by 36.45 mg/dL (95% CI: −65.77, −7.13, *P* = 0.01, I² = 85%). The intervention's effect on Insulin was insignificant, with an effect estimate of −0.12 μU/mL (95% CI: −1.58,1.34) and a heterogeneity I^2^ of 0%, with a *P*-value = 0.87. A sensitivity analysis was not performed due to the low heterogeneity. By subgroup analysis, in one trial with  ≤8 weeks, Insulin changed by −0.19 μU/mL (95% CI: −1.85, 1.47, *P* = 0.82); and in one trial of 8 to 12 weeks, Insulin changed by 0.10 μU/mL (95% CI: −2.96, 3.16, *P* = 0.95). Therefore, the findings of this study do not support the efficacy of the intervention program in reducing insulin levels.

### 3.5. Effects of exercise on body composition

The effects of exercise on metabolic risk included in the systematic review with meta-analysis are presented in Table 2. For RF, the results from eight studies showed that the intervention group had an average reduction of 2.47% in RF (95% CI: −3.42, −1.53, *P* < 0.00001). A sensitivity analysis was not performed due to the low heterogeneity. Subgroup analysis according to the intervention duration showed that in one trial at ≤8 weeks, RF decreased by −2.00% (95% CI: −3.80, −0.20, *P* = 0.03); and in the seven trials at 8 to 12 weeks, RF decreased by −2.65% (95% CI: −3.76, −1.54, *P* < 0.00001, I² = 0%). However, for weight, the results from nine studies showed that the intervention group had an average weight reduction of 0.85 kg (95% CI: −3.48, 1.79, *P* = 0.53) with high heterogeneity (I² = 80%). For the weight outcome measure, heterogeneity I^2^ was 80% with *P* value = 0.53. Due to the high heterogeneity, the sensitivity analysis was performed, and it found that C. Gomez-Tomas, 2018 [50], H. Brain, 2017 [69], L. Macedo Santiago, 2018 [55], and W. H. Son 2023 [58] study may be the source of heterogeneity, possibly due to different interventions. After excluding these studies, the effect was estimated at 0.76 kg (95% CI: −0.33,1.86, *P* = 0.17), and I^2^ was 0%. By subgroup analysis based on intervention duration, in two trials with ≤8 weeks, weight changed by 1.62 kg (95% CI: −5.81, 9.06, *P* = 0.67, I² = 79%); in two trials with 8 to 12 weeks, weight decreased by −2.07 kg (95% CI: −4.24, 0.10, *P* = 0.06, I² = 0%); and in five trials with intervention duration >12 weeks, the weight changed by −1.93 kg (95% CI: −5.56, 1.71, *P* = 0.30, I² = 75%). Regarding waist circumference (WC), the results from four studies showed that the intervention group had an average reduction of 1.96 cm in WC (95% CI: −5.92, 2.00, *P* = 0.33) with heterogeneity of 56%. For WC outcome measures, heterogeneity I^2^ was 56% with *P*-value = 0.33. Due to the low heterogeneity, a sensitivity analysis was performed and found that C. Gomez-Tomas, 2018 [50] and T. C. M., 2018 [59] study may be a source of heterogeneity, possibly due to different control conditions of dietary and lifestyle factors and different measurement methods and time points. After excluding these two articles, the effect estimate was 1.04 cm (95% CI: −2.32,4.41, *P* = 0.54, I^2^ = 0%).By subgroup analysis based on intervention duration, in one trial of ≤8 weeks, WC changed by −1.24 cm (95% CI: −5.99, 3.51, *P* = 0.61); in one trial of 8 to 12 weeks, WC decreased by -5.90  cm (95% CI: −11.07, −0.73, *P* = 0.03); and in two trials with intervention duration >12 weeks, WC changed by −0.79 cm (95% CI: −8.92, 7.34, *P* = 0.85, I² = 77%). Additionally, fat-free mass (FFM) increased by an average of 1.33 kg (95% CI: −0.32, 2.98, *P* = 0.11), and SSM increased by 1.15 kg (95% CI: −0.44, 2.73, *P* = 0.16). Heterogeneity I^2^ was 62% for the FFM outcome measures with a *P* value = 0.11. Due to the high heterogeneity, sensitivity analysis found that A. M. Monteiro, 2022b [67], and S. L. Oh, 2017 [68] may be a source of heterogeneity, which could be differences in training program, training duration, and intensity. After excluding these studies, the effect was estimated at 1.38 kg (95% CI: −0.09,2.85, *P* = 0.07, I^2^ = 0%). In the subgroup of 8- to 12-week intervention durations (two trials), FFM changed by 2.07 kg (95% −0.21, 4.35, *P* = 0.08, I² = 0%); and in the three trials of >12 weeks, FFM changed by 0.93 kg (95% CI: −1.47, 3.34, *P* = 0.45, I² = 78%). For the skeletal muscle mass (SMM) outcome measure, heterogeneity I^2^ was 80% with *P*-value = 0.16. Due to the high heterogeneity, a sensitivity analysis was performed, identifying W. H. Son, 2023 [58] study as a possible source of heterogeneity. After excluding the study, the effect estimate was 1.90 kg (95% CI: 1.05,2.76, *P* < 0.0001, I^2^ was 0%). The reason may be the differences in the training plans and the different characteristics of the study subjects. Subgroup analysis by intervention duration for SMM included three trials with 8 to 12 weeks, showing a change of 1.15 kg (95% CI: −0.44, 2.73, *P* = 0.16, I² = 80%).

The summary results of two studies showed that the average reduction in trunk fat mass (TFM) in the intervention group was 1.72 kg (95% CI: −4.34, 0.90, *P* = 0.20, I² = 71%). Due to the high heterogeneity, a sensitivity analysis was performed, which found that *P*. M. Cunha, 2021b [48] may be a source of heterogeneity, and the reason may be different interventions. After excluding the study, the effect was estimated at −0.56 kg (95% CI: −2.21,1.09, *P* = 0.51, I^2^ = 0%). For subgroup analysis by intervention duration, in two trials with 8 to 12 weeks, TFM changed by −1.72 kg (95% CI: −4.34,0.90, *P* = 0.20, I² = 71%). The results of each subgroup were consistent with the overall outcome trend.

### 3.6. Publication bias

The risk of bias for the included studies is shown in Figures S2 and S3. All included articles reported randomized allocation, with 23 trials describing the specific method of randomization and adopting allocation concealment. All experiments mentioned the reasons for participants' withdrawal or failure to follow up, and 13 experiments were rated as “low risk” due to the principles of intentionality analysis and missing data. 2 experiments only selectively reported the experimental data, and the other risks of bias in the 18 experiments were judged as “unclear.” The overall risk of bias for each included study was judged as “high”. This judgment was primarily due to the inherent challenge of blinding participants and personnel in exercise intervention trials, which led to a high risk of performance bias across all studies.

## 4. Discussion

This meta-analysis, based on randomized controlled trials (RCTs), provides novel insights into the effects of exercise on metabolic risk, cardiovascular health, and body composition in healthy elderly women. Our findings delineate a clear pattern of benefits, underscoring the role of exercise in addressing age-related physiological decline in this demographic.

### 4.1. Metabolic and inflammatory profile

Numerous studies have proved that inflammatory factors spontaneously increase in women after menopause under the influence of ovarian hormones [76,77]. Obesity (dyslipidemia) is associated with long-term increases in inflammatory factors [27,78], such as interleukin 6 (IL-6), tumor necrosis factor *α* (TNF-*α*), and acute phase CRP. Several early biomarker studies have shown that the C-reactive protein is associated with the risk of developing major cardiovascular events and death [79]. Therefore, metabolism, inflammation, and cardiovascular diseases are closely linked. Our analysis, which specifically focuses on elderly women, demonstrates that exercise can significantly reduce CRP levels, extending the understanding of its role in mitigating inflammatory risk in this vulnerable population.

Cholesterol homeostasis is crucial for normal cellular and systemic functions, while dysregulated cholesterol metabolism underlies cardiovascular diseases [80]. Reducing a cholesterol level of about 0.6 mmol/L can reduce the incidence of ischemic heart disease by 54% at age 40% to 19% at age 80 [81]. In a meta-analysis of 170,000 participants, LDL-C reduced the risk of cardiac death, and reducing LDL-C by 40 mg/dL over 5 years reduced the incidence of coronary heart disease events and stroke in all patients and women, and those older than 75 years [82]. Our findings, supported by moderate-certainty evidence, show significant reductions in TC and LDL-C, underscoring the potent lipid-regulating effect of exercise in elderly women. Similarly, reductions in C-reactive protein (CRP), an inflammatory biomarker that independently predicts future vascular events, observed in our analysis (with moderate certainty) align with studies demonstrating that decreased inflammation mediates exercise's cardioprotective effects and predicts a reduced risk of cardiovascular events [28,33].

High-density lipoprotein (HDL) is negatively associated with coronary artery disease (CAD), Higher HDL-C 0 33 mmol/L was associated with about one-third lower ischemic heart disease (IHD) mortality in ischemic heart disease [83]. A cohort study (*n* = 14,478) indicated that extremely high HDL-C levels (>80 mg/dL) were associated with cardiovascular death (HR, 1.71; 95% CI: 1.09–2.68; *P* = 0.02) [84], HDL-C is the only standardized and reproducible parameter that can be used to estimate the plasma concentrations of these lipoproteins [85]. Our data indicate that HDL-C was elevated but insignificant for reducing the adverse cardiovascular risk. Notably, we found no significant change in HDL-C, a finding consistent with resistance training studies in older women and supported by low-certainty evidence in our analysis. This suggests that HDL-C modulation in this population may require longer interventions or combined dietary approaches [26,61].

Our meta-analysis for the outcome of the significant glucose reduction further supports exercise as a cornerstone for metabolic health, likely via improved insulin sensitivity and glucose disposal [86,87]. A population-based, single-center cohort study (*n* = 15,010) found that in addition to a worsened cardiovascular risk profile, diabetic patients observed significant changes in inflammatory and immune responses due to glycemic status [88–91]. We found that the effect on Glu was consistently beneficial during the exercise intervention up to week 12. However, it is important to note that the certainty of evidence for glucose reduction was very low, calling for cautious interpretation. Insulin, as a vasoactive hormone, can regulate the brain and peripheral blood flow; chronic hyperinsulinemia associated with insulin resistance would promote vasoconstriction, leading to increased blood pressure and decreased cerebral perfusion; this pattern may be observed several years before the appearance of the cognitive symptom signature of vascular cognitive impairment [87]. In patients with Alzheimer's disease (AD), cerebrospinal fluid (CSF) concentrations of insulin were reduced, while the plasma concentrations were increased [92]. Our data suggest that although exercise has little effect on the secretory effects of Insulin, our data, based on limited studies and low-certainty evidence, suggest that exercise had little effect on insulin levels.

### 4.2. Cardiovascular fitness

Hypertension is a key risk factor for CVD worldwide [93]. Most married women with a history of hypertension and diabetes mellitus had a high predictive rate of sudden cardiac death [94]. One spans three continents (*n* = 410,000). The meta-analysis shows that Hypertension has a two-fold increased risk of sudden cardiac death (SCD) and SBP for each increase of 20 mmHg, A 28% increase in SCD risk, while no significant association was established between DBP and SCD [95]. The blood pressure reductions observed in our study (with moderate certainty for SBP) are particularly relevant for elderly women, who exhibit higher systolic hypertension prevalence post-menopause due to arterial stiffness exacerbated by estrogen withdrawal [96]. The superior efficacy of 8–12 week interventions aligns with studies showing early-phase vascular adaptations (e.g. endothelial progenitor cell mobilization) peak within this timeframe [97].

Maximum oxygen uptake (VO2peak), sometimes called maximum aerobic capacity or aerobic capacity or aerobic endurance, is a leading indicator of cardiopulmonary fitness [98]. Studies suggest differences in that the mean rate of VO2peak decline in older adults is 4–5 mL kg-1 min-1/per decade [99]. An increase in VO2peak was associated with a decreased risk of all-cause mortality and cardiovascular disease, which was 13% and 15%, respectively [100], primarily through enhanced stroke volume and oxygen extraction capacity [101]. Research suggests that progressive resistance training significantly enhances VO₂max by an average of about 1.8 ml·kg^−1^·min^−1^, with no notable differential effects observed among individuals [102]. These findings indicate that the benefits of resistance training for cardiorespiratory health are both positive and consistent across populations. Our analysis, supported by moderate-certainty evidence, confirms a significant improvement in VO₂peak, underscoring its specific relevance to elderly women, who face unique cardiovascular risks due to aging and hormonal changes.

Regarding HRmax, the peak did not show significant differences after training. The lack of change in maximal heart rate HRmax aligns with cardiac aging physiology: During exercise to exhaustion, increased sympathetic activity and circulating catecholamines together produce exercise intensity-dependent tachycardia; however, the intrinsic heart rate reduction and decreased *β* -adrenergic responsiveness in older individuals restrict the maximal heart rate [103]. This explains why cardiac output improvements primarily derive from stroke volume augmentation rather than heart rate elevation [104].

In summary, the above results are not surprising, as the primary changes during maximal exercise occur in SBP and cardiac output [105].

### 4.3. Body composition

Our meta-analysis revealed a nuanced picture regarding exercise-induced changes in body composition. Some studies have suggested that aging is also associated with a redistribution of fat groups, which preserves body weight and increases WC [106], mainly manifested by an increase in the volume of intra-abdominal adipose tissue (AT) [107]. Some studies have shown that short-term exercise is beneficial for changes in overall fat and biomarkers (visceral abdominal fat and biomarkers), even with weight loss (2% to 6%) [108,109]. This suggests that we need to investigate the effects of longer-term exercise programs on weight. For a given male and female BMI, WC increased by 5 cm, and women increased by 1.13 times (95% CI, 1.11 to 1.15) the mortality risk [110]. However, our meta-analysis failed to elicit statistically significant changes in body weight and WC, outcomes characterized by high heterogeneity and low certainty. This apparent paradox—where beneficial changes in body fat percentage (supported by moderate certainty evidence) occurred without concurrent weight loss—aligns with emerging evidence of "metabolically healthy obesity" phenotypes in aging populations and underscores the importance of body composition remodeling beyond mere weight metrics [111].

Meanwhile, high FFM usually represents low fat mass [112]. Our data showed a nonsignificant increase in the intervention group in FFM. M. E. Nelson shows that for postmenopausal women aged 50–70, the exercise pattern was comparable, with the percentage of body fat decreasing by 1.4% [113]. Our study showed that the significant reduction in RF (with moderate certainty evidence) suggests exercise preferentially mobilizes adipose reserves, and we speculated that exercise had a more significant effect on reducing body fat percentage in older women over 60 years.

The nonsignificant changes in SMM deserve careful interpretation. While pooled analyzes showed minimal gains, previous reviews showed that RT significantly increases muscle mass, with an average of about SMM =  1.1 kg and a wide range of heterogeneity (from 0 to 7.2 kg), with intervention duration ranging from 2 weeks to 1 year [114]. The heterogeneity in muscle outcomes likely stems from variations in training stimulus: low-intensity interventions may inadequately activate muscle protein synthesis pathways in estrogen-deficient older women, who exhibit blunted anabolic responses compared to younger cohorts [115]. This highlights the need for protocol standardization—future interventions should explicitly incorporate progressive overload principles and protein timing strategies to optimize hypertrophy. While our pooled analysis did not show a statistically significant increase in SMM, the observed trend and findings from some individual studies suggest that exercise can positively influence muscle mass. Unlike prior studies that focused on general weight loss or metabolic improvements, our analysis emphasizes the role of exercise in preserving muscle mass and reducing visceral fat, which are critical for preventing sarcopenia and metabolic disorders in this population.

Our findings thus reinforce the "fat quality over fat quantity" paradigm: exercise-induced body composition remodeling confers cardiometabolic protection even in the absence of weight loss.

Subgroup analyzes based on intervention duration suggested that programs lasting 8–12 weeks appeared particularly effective for improving certain outcomes (e.g. VO₂peak, TG, RF). However, the high heterogeneity observed in some longer-term (>12 weeks) outcomes (e.g. weight, WC) may be partly explained by the considerable variation in exercise intensity, supervision, and adherence across these studies, as detailed in Supplementary Table S2. Future studies should employ more standardized and comprehensively reported exercise prescriptions to better elucidate the dose‒response relationship.

### 4.4. Limitations and strengths

While our findings offer practical insights into the overall benefits of structured exercise, several limitations should be considered when interpreting the results. First, the observed clinical heterogeneity in exercise protocols, combined with an insufficient number of studies for each modality, precluded a formal subgroup analysis to compare different types of exercise directly. This is a significant limitation that future research should aim to address. Second, the potential acute effects of exercise on inflammatory markers such as CRP might introduce additional variability. Third, the interpretation of several outcomes (e.g. Glu, CRP, weight, WC, FFM, and SMM) is tempered by their low certainty of evidence according to the GRADE framework and the potential for publication bias inherent in meta-analyzes with a limited number of studies. We addressed this specifically through a qualitative assessment of gray literature (as detailed in Section 2.6), rather than relying on underpowered statistical tests.

Despite these limitations, we believe this meta-analysis provides a meaningful synthesis of the available RCT evidence regarding exercise interventions in elderly women. The consistency observed across sensitivity and subgroup analyzes—particularly with respect to intervention duration—strengthens confidence in the main findings. Our study thus offers a foundational evidence base to inform the design of combined exercise programs in this population, while also highlighting the need for more standardized, modality-specific trials in the future.

### 4.5. Future perspectives

Exercise in older women may gain significant protection from the harmful effects of excess body fat, metabolic risk, and inflammation. However, we should also make some observations for future studies. First, non-English literature can be included to reduce publication bias, and second, priority control of dietary intake and physical activity levels during the intervention can be used to ensure that adaptive responses are not affected by confounding variables. Third, more cardiovascular indicators should be analyzed because there is insufficient data for the meta-analysis of many outcomes.

## 5. Conclusion

In conclusion, this meta-analysis demonstrates that exercise interventions in older women lead to significant improvements in several key health indicators. Based on moderate certainty evidence, exercise reduces lipid levels (TG, TC) and relative body fat. Supported by moderate certainty evidence, it also lowers systolic blood pressure while enhancing VO2peak. These adaptations are critical for mitigating age-related metabolic dysregulation and lowering cardiovascular risk. While significant changes in body weight and waist circumference were not observed (findings limited by low certainty evidence), the favorable remodeling of body composition and improvement in metabolic and inflammatory profiles are pivotal for potentially delaying aging processes and reducing cardiovascular events. Future studies should investigate the combined effects of exercise and other lifestyle interventions (e.g. dietary modification, sleep improvement, etc.) to provide a more holistic health promotion strategy.

## Supplementary Material

Supplementary Material Supplementary_Figureclean

Supplementary Material Supplementary_Tabless.docx

## Data Availability

Data supporting this study are included within the article and supporting materials.

## References

[1] Kanasi E , Ayilavarapu S , Jones J . The aging population: demographics and the biology of aging. Periodontol 2000. 2016 Oct;72(1):13–18. doi: 10.1111/prd.12126 27501488

[2] Ihalainen JK , Inglis A , Mäkinen T , et al. Strength training improves metabolic health markers in older individual regardless of training frequency. Front Physiol. 2019;10:32. doi: 10.3389/fphys.2019.00032 30774600 PMC6367240

[3] Zamboni M , Rossi AP , Fantin F , et al. Adipose tissue, diet and aging. Mech Ageing Dev. 2014 Mar-Apr;136-137:129–137. doi: 10.1016/j.mad.2013.11.008 24321378

[4] Son WH , Park HT , Jeon BH , et al. Moderate intensity walking exercises reduce the body mass index and vascular inflammatory factors in postmenopausal women with obesity: a randomized controlled trial. Sci Rep. 2023 Nov 17;13(1):20172. doi: 10.1038/s41598-023-47403-2 37978254 PMC10656478

[5] Alghadir AH , Gabr SA , Al-Eisa ES . Effects of moderate aerobic exercise on cognitive abilities and redox state biomarkers in older adults. Oxid Med Cell Longev. 2016;2016:2545168. doi: 10.1155/2016/2545168 27195073 PMC4852338

[6] Stamatakis E , Ahmadi M , Biswas RK , et al. Device-measured vigorous intermittent lifestyle physical activity (VILPA) and major adverse cardiovascular events: evidence of sex differences. Br J Sports Med. 2025 Feb 20;59(5):316–324. doi: 10.1136/bjsports-2024-108484 39467622 PMC11874358

[7] Mann S , Beedie C , Jimenez A . Differential effects of aerobic exercise, resistance training and combined exercise modalities on cholesterol and the lipid profile: review, synthesis and recommendations. Sports Med. 2014 Feb;44(2):211–221. doi: 10.1007/s40279-013-0110-5 24174305 PMC3906547

[8] American College of Sports Medicine position stand . Progression models in resistance training for healthy adults. Med Sci Sports Exerc. 2009 Mar;41(3):687–708. doi: 10.1249/MSS.0b013e3181915670 19204579

[9] Fragala MS , Cadore EL , Dorgo S , et al. Resistance training for older adults: position statement from The National strength and conditioning association. J Strength Cond Res. 2019 Aug;33(8):2019–2052. doi: 10.1519/JSC.0000000000003230 31343601

[10] Holloszy JO , Coyle EF . Adaptations of skeletal muscle to endurance exercise and their metabolic consequences. J Appl Physiol Respir Environ Exerc Physiol. 1984 Apr;56(4):831–838. doi: 10.1152/jappl.1984.56.4.831 6373687

[11] Thompson PD , Arena R , Riebe D , et al. ACSM's new preparticipation health screening recommendations from ACSM's guidelines for exercise testing and prescription, ninth edition. Curr Sports Med Rep. 2013 Jul-Aug;12(4):215–217. doi: 10.1249/JSR.0b013e31829a68cf 23851406

[12] Peterson MD , Rhea MR , Sen A , et al. Resistance exercise for muscular strength in older adults: a meta-analysis. #N/A. 2010 Jul;9(3):226–237. 10.1016/j.arr.2010.03.004 PMC2892859 20385254

[13] Westcott WL . Resistance training is Medicine: effects of strength training on health. Curr Sports Med Rep. 2012 Jul-Aug;11(4):209–216. doi: 10.1249/JSR.0b013e31825dabb8 22777332

[14] Linhares DG , Borba-Pinheiro CJ , Castro JBP , et al. Effects of multicomponent exercise training on the health of older women with osteoporosis: a systematic review and meta-analysis. Int J Env Res Public Health. 2022 Oct 30;19(21):14195. doi: 10.3390/ijerph192114195 36361073 PMC9655411

[15] Sallinen J , Pakarinen A , Fogelholm M , et al. Serum basal hormone concentrations and muscle mass in aging women: effects of strength training and diet. Int J Sport Nutr Exerc Metab. 2006;16(3):316–331. doi: 10.1123/ijsnem.16.3.316 16948487

[16] Walker S , Peltonen H , Sautel J , et al. Neuromuscular adaptations to constant vs. Variable resistance training in older men. Int J Sports Med. 2013;35(1):69–74. doi: 10.1055/s-0033-1343404 23825004

[17] Tomeleri CM , Ribeiro AS , Souza MF , et al. Resistance training improves inflammatory level, lipid and glycemic profiles in obese older women: a randomized controlled trial. Exp Gerontol. 2016 Nov;84:80–87. doi: 10.1016/j.exger.2016.09.005 27616162

[18] Karvonen-Gutierrez C , Kim C . Association of mid-life changes in body size, body composition and obesity status with the menopausal transition. Healthcare (Basel). 2016 Jul 13;4(3):42. doi: 10.3390/healthcare4030042 27417630 PMC5041043

[19] Zhou M , Zhao G , Zeng Y , et al. Aging and cardiovascular disease: current status and challenges. Rev Cardiovasc Med. 2022 Apr;23(4):135. doi: 10.31083/j.rcm2304135 39076212 PMC11274005

[20] Callow AD . Cardiovascular disease 2005--the global picture. Vascul Pharmacol. 2006 Nov;45(5):302–307. doi: 10.1016/j.vph.2006.08.010 17074537

[21] Appelman Y , van Rijn BB , Ten Haaf ME , et al. Sex differences in cardiovascular risk factors and disease prevention. Atherosclerosis. 2015 Jul;241(1):211–218. doi: 10.1016/j.atherosclerosis.2015.01.027 25670232

[22] Bellettiere J , LaMonte MJ , Evenson KR , et al. Sedentary behavior and cardiovascular disease in older women: the objective physical activity and cardiovascular health (OPACH) study. Circulation. 2019 Feb 19;139(8):1036–1046. doi: 10.1161/CIRCULATIONAHA.118.035312 31031411 PMC6481298

[23] Expert Panel on Detection, Evaluation, and Treatment of High Blood Cholesterol in adults. Executive summary of the third report of The National cholesterol education program (NCEP) expert panel on detection, evaluation, and treatment of high blood cholesterol in adults (Adult Treatment Panel III). JAMA. 2001 May 16;285(19):2486–2497. doi: 10.1001/jama.285.19.2486 11368702

[24] Malik S , Wong ND , Franklin SS , et al. Impact of the metabolic syndrome on mortality from coronary heart disease, cardiovascular disease, and all causes in United States adults. Circulation. 2004 Sep 7;110(10):1245–1250. doi: 10.1161/01.CIR.0000140677.20606.0E 15326067

[25] Tchernof A , Després JP . Pathophysiology of human visceral obesity: an update. Physiol Rev. 2013 Jan;93(1):359–404. doi: 10.1152/physrev.00033.2011 23303913

[26] Porter RR , Sparks JR , Durstine JL , et al. Effect of exercise training on lipoprotein subclass particle concentrations and sizes in older women: results from a randomized controlled trial. Geriatrics (Basel). 2023 Nov 29;8(6):116. doi: 10.3390/geriatrics8060116 38132487 PMC10742846

[27] Kim JK , Moon DW , Chung YT , et al. The factors associated with high-sensitivity C-Reactive protein in postmenopausal women: based on korea national health and nutrition examination survey 2016–2017. Korean Journal of Family Practice. 2020;10(2):96–102. doi: 10.21215/kjfp.2020.10.2.96

[28] Moriya J . Critical roles of inflammation in atherosclerosis. J Cardiol. 2019 Jan;73(1):22–27. doi: 10.1016/j.jjcc.2018.05.010 29907363

[29] Ellulu MS , Patimah I , Khaza'ai H , et al. Obesity and inflammation: the linking mechanism and the complications. Arch Med Sci. 2017 Jun;13(4):851–863. doi: 10.5114/aoms.2016.58928 28721154 PMC5507106

[30] Nejatian Hoseinpour A , Bassami M , Ahmadizad S , et al. The influence of resistance training on inflammatory markers, body composition and functional capacity in healthy older adults: a systematic review and meta-analysis. Arch Gerontol Geriatr. 2025 Mar;130:105731. doi: 10.1016/j.archger.2024.105731 39740358

[31] Monteiro-Junior RS , de Tarso Maciel-Pinheiro P , da Matta Mello Portugal E , et al. Effect of exercise on inflammatory profile of older persons: systematic review and meta-analyses. J Phys Act Health. 2018 Jan 1;15(1):64–71. doi: 10.1123/jpah.2016-0735 28771081

[32] Abd El-Kader SM , Al-Shreef FM , Al-Jiffri OH . Impact of aerobic exercise versus resisted exercise on endothelial activation markers and inflammatory cytokines among elderly. Afr Health Sci. 2019 Dec;19(4):2874–2880. doi: 10.4314/ahs.v19i4.9 32127863 PMC7040351

[33] Loaiza-Betancur AF , Gómez-Tomás C , Blasco JM , et al. Effects of resistance training on C-reactive protein in menopausal and postmenopausal women: a systematic review and meta-analysis of randomized controlled trials. The North American Menopause Society. 2022 Dec 1;29(12):1430–1440. doi: 10.1097/GME.0000000000002076 36219807

[34] Zheng G , Qiu P , Xia R , et al. Effect of aerobic exercise on inflammatory markers in healthy middle-aged and older adults: a systematic review and meta-analysis of randomized controlled trials. Front Aging Neurosci. 2019;11:98. doi: 10.3389/fnagi.2019.00098 31080412 PMC6497785

[35] Grant BF , Chou SP , Saha TD , et al. Prevalence of 12-Month alcohol use, high-risk drinking, and DSM-IV alcohol use disorder in the United States, 2001-2002 to 2012-2013: results from The National epidemiologic survey on alcohol and related conditions. #N/A. 2017 Sep 1;74(9):911–923. 10.1001/jamapsychiatry.2017.2161 PMC5710229 28793133

[36] Wang TW , Asman K , Gentzke AS , et al. Tobacco product use among adults - United States, 2017. MMWR Morb Mortal Wkly Rep. 2018 Nov 9;67(44):1225–1232. doi: 10.15585/mmwr.mm6744a2 30408019 PMC6223953

[37] Chodzko-Zajko WJ , Proctor DN , Fiatarone Singh MA , et al. American college of sports Medicine position stand. Exercise and physical activity for older adults. Med Sci Sports Exerc. 2009 Jul;41(7):1510–1530. doi: 10.1249/MSS.0b013e3181a0c95c 19516148

[38] Slade SC , Dionne CE , Underwood M , et al. Consensus on exercise reporting template (CERT): explanation and elaboration statement. Br J Sports Med. 2016 Dec;50(23):1428–1437. doi: 10.1136/bjsports-2016-096651 27707738

[39] Troiano RP , Berrigan D , Dodd KW , et al. Physical activity in the United States measured by accelerometer. Med Sci Sports Exerc. 2008 Jan;40(1):181–188. doi: 10.1249/mss.0b013e31815a51b3 18091006

[40] Füzéki E , Engeroff T , Banzer W . Health benefits of light-intensity physical activity: a systematic review of accelerometer data of The National health and nutrition examination survey (NHANES). Sports Med. 2017 Sep;47(9):1769–1793. doi: 10.1007/s40279-017-0724-0 28393328

[41] Piercy KL , Troiano RP , Ballard RM , et al. The physical activity guidelines for Americans. #N/A. 2018 Nov 20;320(19):2020–2028. 10.1001/jama.2018.14854 PMC9582631 30418471

[42] Bull FC , Al-Ansari SS , Biddle S , et al. World health organization 2020 guidelines on physical activity and sedentary behaviour. Br J Sports Med. 2020 Dec;54(24):1451–1462. doi: 10.1136/bjsports-2020-102955 33239350 PMC7719906

[43] Page MJ , McKenzie JE , Bossuyt PM , et al. The PRISMA 2020 statement: an updated guideline for reporting systematic reviews. BMJ. 2021 Mar 29;372:n71. doi: 10.1136/bmj.n71 33782057 PMC8005924

[44] United Nations DoEaSA . World Population Ageing 2015. New York, NY: Department of Economic and Social Affairs; 2015.

[45] Ainsworth BE , Haskell WL , Herrmann SD , et al. 2011 compendium of physical activities: a second update of codes and MET values. Med Sci Sports Exerc. 2011 Aug;43(8):1575–1581. doi: 10.1249/MSS.0b013e31821ece12 21681120

[46] Garber CE , Blissmer B , Deschenes MR , et al. American college of sports Medicine position stand. Quantity and quality of exercise for developing and maintaining cardiorespiratory, musculoskeletal, and neuromotor fitness in apparently healthy adults: guidance for prescribing exercise. Med Sci Sports Exerc. 2011 Jul;43(7):1334–1359. doi: 10.1249/MSS.0b013e318213fefb 21694556

[47] Innovation VH . Covidence systematic review software Melbourne. Australia: Veritas Health Innovation; 2025. n.d. [15.03.2025]. Available from: https://www.covidence.org/

[48] Cunha PM , Tomeleri CM , Nascimento MA , et al. Comparision of low and high volume of resistance training on body fat and blood biomarkers in untrained older women: a randomized clinical trial [Article]. J Strength Cond Res. 2021;35(1):1–8. doi: 10.1519/JSC.0000000000003245 31306389

[49] Tomeleri CM , Ribeiro AS , Cavaglieri CR , et al. Correlations between resistance training-induced changes on phase angle and biochemical markers in older women [Article]. Scand J Med Sci Sports. 2018;28(10):2173–2182. doi: 10.1111/sms.13232 29858504

[50] Gómez-Tomás C , Chulvi-Medrano I , José Carrasco J , et al. Effect of a 1-year elastic band resistance exercise program on cardiovascular risk profile in postmenopausal women [Article]. The North American Menopause Society. 2018;25(9):1004–1010. doi: 10.1097/GME.0000000000001113 29787478

[51] de Souza HCM , Pessoa MF , Clemente RDS , et al. Effects of 12 weeks of inspiratory muscle training and whole body vibration on the inflammatory profile, BDNF and muscular system in pre-frail elderly women: a randomized controlled trial [Article]. Arch Gerontol Geriatr. 2024;123:105421. doi: 10.1016/j.archger.2024.105421 38593699

[52] Stojanović MDM , Mikić MJ , Milošević Z , et al. Effects of chair-based, low-load elastic band resistance training on functional fitness and metabolic biomarkers in older women [Article]. Journal of sports science & medicine. 2021;20(1):133–141. doi: 10.52082/jssm.2021.133 33707996 PMC7919366

[53] Rodrigues-Krause J , Farinha JB , Ramis TR , et al. Effects of dancing compared to walking on cardiovascular risk and functional capacity of older women: a randomized controlled trial [Article]. Exp Gerontol. 2018;114:67–77. doi: 10.1016/j.exger.2018.10.015 30389581

[54] Tomeleri CM , Cunha PM , Dib MM , et al. Effect of resistance exercise order on cardiovascular disease risk factors in older women: a randomized controlled trial [Article]. Int J Env Res Public Health. 2023;20(2):1165. doi: 10.3390/ijerph20021165 36673920 PMC9859374

[55] Macêdo Santiago L , Neto LGL , Borges Pereira G , et al. Effects of resistance training on immunoinflammatory response, TNF-Alpha gene expression, and body composition in elderly women. J Aging Res. 2018;2018:1467025. doi: 10.1155/2018/1467025 30510801 PMC6230406

[56] Elsangedy HM , Oliveira GTA , Machado D , et al. Effects of self-selected resistance training on physical fitness and psychophysiological responses in physically inactive older women: a randomized controlled study. Percept Motor Skills. 2021 Feb;128(1):467–491. doi: 10.1177/0031512520967610 33115322

[57] Kortas J , Ziemann E , Juszczak D , et al. Iron status in elderly women impacts myostatin, adiponectin and osteocalcin levels induced by nordic walking training [Article]. the Nutrients. 2020;12(4):1129. doi: 10.3390/nu12041129 32316589 PMC7231223

[58] Son WH , Park HT , Jeon BH , et al. Moderate intensity walking exercises reduce the body mass index and vascular inflammatory factors in postmenopausal women with obesity: a randomized controlled trial [Article]. Sci Rep. 2023;13(1):20172. doi: 10.1038/s41598-023-47403-2 37978254 PMC10656478

[59] Tomeleri CM , Ribeiro AS , Souza MF , et al. Resistance training reduces metabolic syndrome and inflammatory markers in older women: a randomized controlled trial. J Diabetes. 2018;10(4):328–337. doi: 10.1111/1753-0407.12614 29031002

[60] Teixeira do Amaral V , Zanini GS , Marçal IR , et al. Superior effect of long-term community-based high-intensity interval training on cardiovascular and functional parameters in low-income older women [Article]. the European Journal of Preventive Cardiology. 2024;31(12):1543–1546. doi: 10.1093/eurjpc/zwae200 38864467

[61] Costa RR , Buttelli ACK , Coconcelli L , et al. Water-based aerobic and resistance training as a treatment to improve the lipid profile of women with dyslipidemia: a randomized controlled trial. Journal of Physical Activity and Health. 2019 May;16(5):348–354. doi: 10.1123/jpah.2018-0602 30991881

[62] Costa RR , Kanitz AC , Reichert T , et al. Water-based aerobic training improves strength parameters and cardiorespiratory outcomes in elderly women [Article]. Exp Gerontol. 2018;108:231–239. doi: 10.1016/j.exger.2018.04.022 29730330

[63] Andrade LS , Pinto SS , Silva MR , et al. Water-based continuous and interval training in older women: cardiorespiratory and neuromuscular outcomes (WATER study) [Article]. Exp Gerontol. 2020;134:110914. doi: 10.1016/j.exger.2020.110914 32145293

[64] Häfele MS , Alberton CL , Häfele V , et al. Water-based training programs improve functional capacity, cognitive and hemodynamic outcomes? The ACTIVE randomized clinical trial [Article]. Res Q Exerc Sport. 2023;94(1):24–34. doi: 10.1080/02701367.2021.1935433 35294330

[65] Carrasco-Poyatos M , Rubio-Arias JA , Ballesta-García I , et al. Pilates vs. Muscular training in older women. Effects in functional factors and the cognitive interaction: a randomized controlled trial. Physiol Behav. 2019 Mar 15;201:157–164. doi: 10.1016/j.physbeh.2018.12.008 30529737

[66] Urzi F , Marusic U , Ličen S , et al. Effects of elastic resistance training on functional performance and myokines in older Women-A randomized controlled trial. J Am Med Dir Assoc. 2019 Jul;20(7):830–834.e2. doi: 10.1016/j.jamda.2019.01.151 30902674

[67] Monteiro AM , Rodrigues S , Matos S , et al. The effects of 32 weeks of multicomponent training with different exercises order in elderly Women's functional fitness and body composition. Medicina (Kaunas). 2022 Apr 30;58(5):628. doi: 10.3390/medicina58050628 35630045 PMC9146974

[68] Oh SL , Kim HJ , Woo S , et al. Effects of an integrated health education and elastic band resistance training program on physical function and muscle strength in community-dwelling elderly women: healthy aging and happy aging II study. Geriatr Gerontol Int. 2017 May;17(5):825–833. doi: 10.1111/ggi.12795 27138245

[69] Blain H , Jaussent A , Picot MC , et al. Effect of a 6-Month brisk walking program on walking endurance in sedentary and physically deconditioned women aged 60 or older: a randomized trial. Journal Of Nutrition Health and Aging. 2017;21(10):1183–1189. doi: 10.1007/s12603-017-0955-7 PMC12880060 29188878

[70] Sterne JAC , Savović J , Page MJ , et al. RoB 2: a revised tool for assessing risk of bias in randomised trials. BMJ. 2019 Aug 28;366:l4898. doi: 10.1136/bmj.l4898 31462531

[71] Schünemann JPH HJ , Vist GE . Completing ‘Summary of findings’ tables and grading the certainty of the evidence In: Cochrane Handbook for Systematic Reviews of Interventions. Hoboken, NJ: John Wiley & Sons Ltd; 2019. pp. 375–402. doi: 10.1002/9781119536604.ch14

[72] Higgins JPTS , E , Li T . Chapter 23: including variants on randomized trials In: Cochrane Handbook for Systematic Reviews of Interventions Version 6.4. Version 6.4 (Updated August 2023) ed. London, UK: Cochrane; 2023.

[73] Duval S , Tweedie R . Trim and fill: a simple funnel-plot-based method of testing and adjusting for publication bias in meta-analysis. Biometrics. 2000 Jun;56(2):455–463. doi: 10.1111/j.0006-341X.2000.00455.x 10877304

[74] Shi L , Lin L . The trim-and-fill method for publication bias: practical guidelines and recommendations based on a large database of meta-analyses. Medicine (Baltimore). 2019 Jun;98(23):e15987. doi: 10.1097/MD.0000000000015987 31169736 PMC6571372

[75] Peters JL , Sutton AJ , Jones DR , et al. Performance of the trim and fill method in the presence of publication bias and between-study heterogeneity. Stat Med. 2007 Nov 10;26(25):4544–4562. doi: 10.1002/sim.2889 17476644

[76] Pfeilschifter J , Köditz R , Pfohl M , et al. Changes in proinflammatory cytokine activity after menopause. Endocr Rev. 2002 Feb;23(1):90–119. doi: 10.1210/edrv.23.1.0456 11844745

[77] Mumusoglu S , Yildiz BO . Metabolic syndrome during menopause. Curr Vasc Pharmacol. 2019;17(6):595–603. doi: 10.2174/1570161116666180904094149 30179134

[78] Tilg H , Moschen AR . Adipocytokines: mediators linking adipose tissue, inflammation and immunity. Nature Reviews. 2006 Oct;6(10):772–783. doi: 10.1038/nri1937 16998510

[79] Andersson C , Nayor M , Tsao CW , et al. Framingham heart study: JACC focus seminar, 1/8. J Am Coll Cardiol. 2021 Jun 1;77(21):2680–2692. doi: 10.1016/j.jacc.2021.01.059 34045026

[80] Luo J , Yang H , Song BL . Mechanisms and regulation of cholesterol homeostasis. The Nature Reviews Molecular Cell Biology. 2020 Apr;21(4):225–245. doi: 10.1038/s41580-019-0190-7 31848472

[81] Law MR , Wald NJ , Thompson SG . By how much and how quickly does reduction in serum cholesterol concentration lower risk of ischaemic heart disease? BMJ. 1994 Feb 5;308(6925):367–372. doi: 10.1136/bmj.308.6925.367 8043072 PMC2539460

[82] Smith DA . ACP journal club. Meta-analysis: more intensive statin therapy prevents major vascular events. Ann Intern Med. 2011 May 17;154(10):Jc5–03. doi: 10.7326/0003-4819-154-10-201105170-02003 21576521

[83] Lewington S , Whitlock G , Clarke R , et al. Blood cholesterol and vascular mortality by age, sex, and blood pressure: a meta-analysis of individual data from 61 prospective studies with 55,000 vascular deaths. Lancet. 2007 Dec 1;370(9602):1829–1839. doi: 10.1016/S0140-6736(07)61778-4 18061058

[84] Liu C , Dhindsa D , Almuwaqqat Z , et al. Association between high-density lipoprotein cholesterol levels and adverse cardiovascular outcomes in high-risk populations. JAMA Cardiol. 2022 Jul 1;7(7):672–680. doi: 10.1001/jamacardio.2022.0912 35583863 PMC9118072

[85] Pérez-Méndez Ó , Pacheco HG , Martínez-Sánchez C , et al. HDL-cholesterol in coronary artery disease risk: function or structure? Clin Chim Acta. 2014 Feb 15;429:111–122. doi: 10.1016/j.cca.2013.12.001 24333390

[86] Colberg SR , Sigal RJ , Fernhall B , et al. Exercise and type 2 diabetes: the American college of sports Medicine and the American diabetes association: joint position statement. Diabetes Care. 2010 Dec;33(12):e147–67. doi: 10.2337/dc10-9990 21115758 PMC2992225

[87] Kellar D , Craft S . Brain insulin resistance in Alzheimer's disease and related disorders: mechanisms and therapeutic approaches. Lancet Neurol. 2020 Sep;19(9):758–766. doi: 10.1016/S1474-4422(20)30231-3 32730766 PMC9661919

[88] Donath MY , Shoelson SE . Type 2 diabetes as an inflammatory disease. Nature Reviews. 2011 Feb;11(2):98–107. doi: 10.1038/nri2925 21233852

[89] Pradhan AD , Manson JE , Rifai N , et al. C-reactive protein, interleukin 6, and risk of developing type 2 diabetes mellitus. JAMA. 2001 Jul 18;286(3):327–334. doi: 10.1001/jama.286.3.327 11466099

[90] Luotola K , Pietilä A , Zeller T , et al. Associations between interleukin-1 (IL-1) gene variations or IL-1 receptor antagonist levels and the development of type 2 diabetes. J Intern Med. 2011 Mar;269(3):322–332. doi: 10.1111/j.1365-2796.2010.02294.x 21205020

[91] Grossmann V , Schmitt VH , Zeller T , et al. Profile of the immune and inflammatory response in individuals with prediabetes and type 2 diabetes. Diabetes Care. 2015 Jul;38(7):1356–1364. doi: 10.2337/dc14-3008 25877811

[92] BlÃ¡zquez E , Velázquez E , Hurtado-Carneiro V , et al. Insulin in the brain: its pathophysiological implications for states related with central insulin resistance, type 2 diabetes and Alzheimer's disease. Front Endocrinol (Lausanne). 2014;5:161. doi: 10.3389/fendo.2014.00161 25346723 PMC4191295

[93] Bromfield S , Muntner P . High blood pressure: the leading global burden of disease risk factor and the need for worldwide prevention programs. Curr Hypertens Rep. 2013 Jun;15(3):134–136. doi: 10.1007/s11906-013-0340-9 23536128 PMC3699411

[94] Belhaj A , Shimi M , Kort I , et al. Risk factors of sudden cardiac death in women: a 10 years study in Tunisia. J Forensic Leg Med. 2023 May;96:102517. doi: 10.1016/j.jflm.2023.102517 37004373

[95] Pan H , Hibino M , Kobeissi E , et al. Blood pressure, hypertension and the risk of sudden cardiac death: a systematic review and meta-analysis of cohort studies. Eur J Epidemiol. 2020 May;35(5):443–454. doi: 10.1007/s10654-019-00593-4 31875269 PMC7250808

[96] Maas AH , Franke HR . Women's health in menopause with a focus on hypertension. Neth Heart J. 2009 Feb;17(2):68–72. doi: 10.1007/BF03086220 19247469 PMC2644382

[97] Tilling L , Chowienczyk P , Clapp B . Progenitors in motion: mechanisms of mobilization of endothelial progenitor cells. Br J Clin Pharmacol. 2009 Oct;68(4):484–492. doi: 10.1111/j.1365-2125.2009.03486.x 19843051 PMC2780273

[98] Srivastava S , Tamrakar S , Nallathambi N , et al. Assessment of maximal oxygen uptake (VO2 Max) in athletes and nonathletes assessed in sports physiology laboratory. Cureus. 2024 May;16(5):e61124. doi: 10.7759/cureus.61124 38919211 PMC11197041

[99] Shephard RJ . Maximal oxygen intake and independence in old age. Br J Sports Med. 2009 May;43(5):342–346. doi: 10.1136/bjsm.2007.044800 18403414

[100] Kodama S , Saito K , Tanaka S , et al. Cardiorespiratory fitness as a quantitative predictor of all-cause mortality and cardiovascular events in healthy men and women: a meta-analysis. JAMA. 2009 May 20;301(19):2024–2035. doi: 10.1001/jama.2009.681 19454641

[101] Shibata S , Fujimoto N , Hastings JL , et al. The effect of lifelong exercise frequency on arterial stiffness. J Physiol. 2018 Jul;596(14):2783–2795. doi: 10.1113/JP275301 29781119 PMC6046080

[102] Kelley GA , Kelley KS , Stauffer BL . Resistance training and inter-interindividual response differences on cardiorespiratory fitness in older adults: an ancillary meta-analysis of randomized controlled trials. Sci Prog. 2024 Jan-Mar;107(1):368504241227088. doi: 10.1177/00368504241227088 38312013 PMC10846148

[103] Christou DD , Seals DR . Decreased maximal heart rate with aging is related to reduced {beta}-adrenergic responsiveness but is largely explained by a reduction in intrinsic heart rate. J Appl Physiol (1985). 2008 Jul;105(1):24–29. doi: 10.1152/japplphysiol.90401.2008 18483165 PMC2494835

[104] Ozemek C , Whaley MH , Finch WH , et al. Maximal heart rate declines linearly with age independent of cardiorespiratory fitness levels. #N/A. 2017 Jun;17(5):563–570. 10.1080/17461391.2016.1275042 28099086

[105] Powers ETH SK . Exercise Physiology: Theory and Application to Fitness and Performance. 9 ed. New York: McGraw-Hill Education; 2015.

[106] Fantin F , Di Francesco V , Fontana G , et al. Longitudinal body composition changes in old men and women: interrelationships with worsening disability. J Gerontol A Biol Sci Med Sci. 2007 Dec;62(12):1375–1381. doi: 10.1093/gerona/62.12.1375 18166688

[107] Kotani K , Tokunaga K , Fujioka S , et al. Sexual dimorphism of age-related changes in whole-body fat distribution in the obese. Int J Obes Relat Metab Disord. 1994 Apr;18(4):207–2. 8044194

[108] Donnelly JE , Blair SN , Jakicic JM , et al. American college of sports Medicine position stand. Appropriate physical activity intervention strategies for weight loss and prevention of weight regain for adults. Med Sci Sports Exerc. 2009 Feb;41(2):459–471. doi: 10.1249/MSS.0b013e3181949333 19127177

[109] Kay SJ , Fiatarone Singh MA . The influence of physical activity on abdominal fat: a systematic review of the literature. Obes Rev. 2006 May;7(2):183–200. doi: 10.1111/j.1467-789X.2006.00250.x 16629874

[110] Pischon T , Boeing H , Hoffmann K , et al. General and abdominal adiposity and risk of death in Europe. New Engl J Med. 2008 Nov 13;359(20):2105–2120. doi: 10.1056/NEJMoa0801891 19005195

[111] Bowman K , Atkins JL , Delgado J , et al. Central adiposity and the overweight risk paradox in aging: follow-up of 130,473 UK biobank participants. AJCN. 2017 Jul;106(1):130–135. doi: 10.3945/ajcn.116.147157 PMC5486197 28566307

[112] Oliver CJ , Climstein M , Rosic N , et al. Fat-free mass: friend or foe to metabolic health? J Cachexia Sarcopenia Muscle. 2025 Feb;16(1):e13714. doi: 10.1002/jcsm.13714 39895188 PMC11788497

[113] Nelson ME , Fiatarone MA , Morganti CM , et al. Effects of high-intensity strength training on multiple risk factors for osteoporotic fractures. A randomized controlled trial. JAMA. 1994 Dec 28;272(24):1909–1914. doi: 10.1001/jama.1994.03520240037038 7990242

[114] Benito PJ , Cupeiro R , Ramos-Campo DJ , et al. A systematic review with meta-analysis of the effect of resistance training on whole-body muscle growth in healthy adult males. Int J Env Res Public Health. 2020 Feb 17;17(4):1285. doi: 10.3390/ijerph17041285 32079265 PMC7068252

[115] Orsatti FL , Nunes PRP , Carneiro M , et al. Heterogeneity in resistance training-induced muscle strength responses is associated with training frequency and insulin resistance in postmenopausal women. Exp Gerontol. 2022 Jun 15;163:111807. doi: 10.1016/j.exger.2022.111807 35421558

