# Supplementary material for: Effects of exercise on metabolic risk, cardiovascular fitness, and body composition in elderly women of the past decade: a systematic review and meta-analysis
Source: J Int Soc Sports Nutr. 2026 Jun 2;23(1):2675444. doi: 10.1080/15502783.2026.2675444 (PMC13231817; doi:10.1080/15502783.2026.2675444)

**Supplementary Figure**

Fig S1. Forest Plot of subgroups.

HRmax


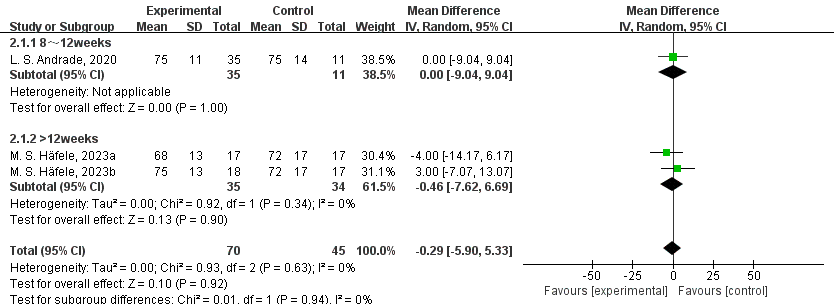


VO2peak


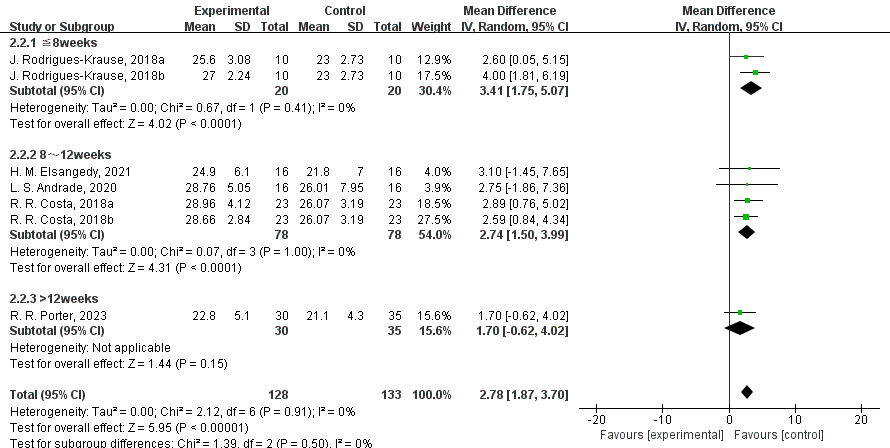


SBP


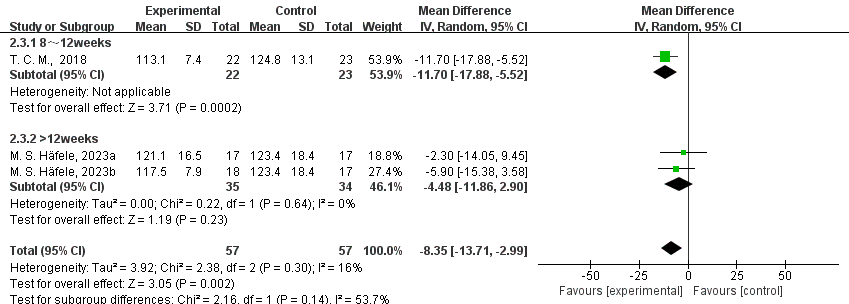


DBP


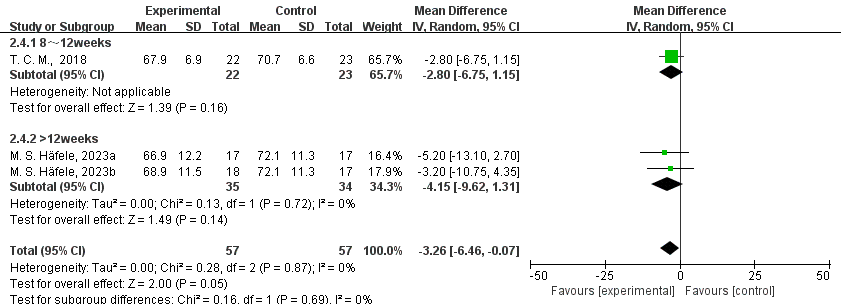


TG


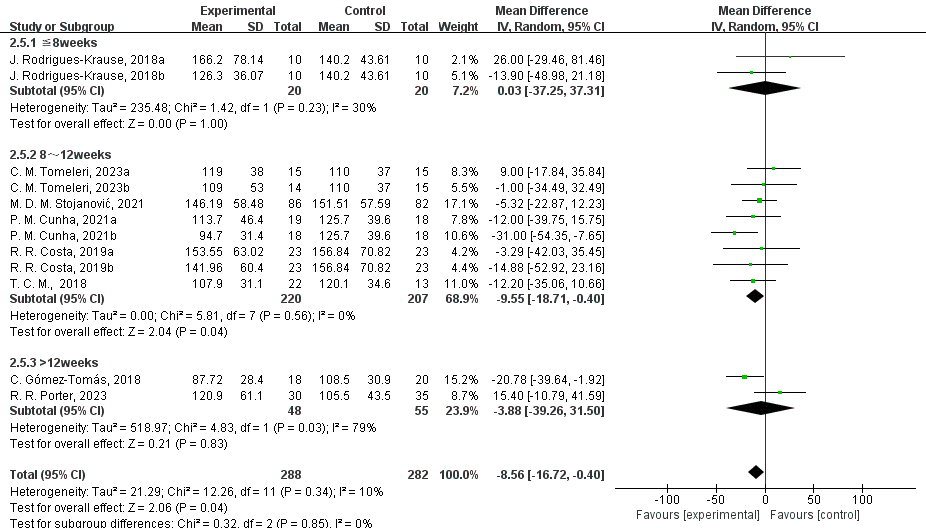


TC


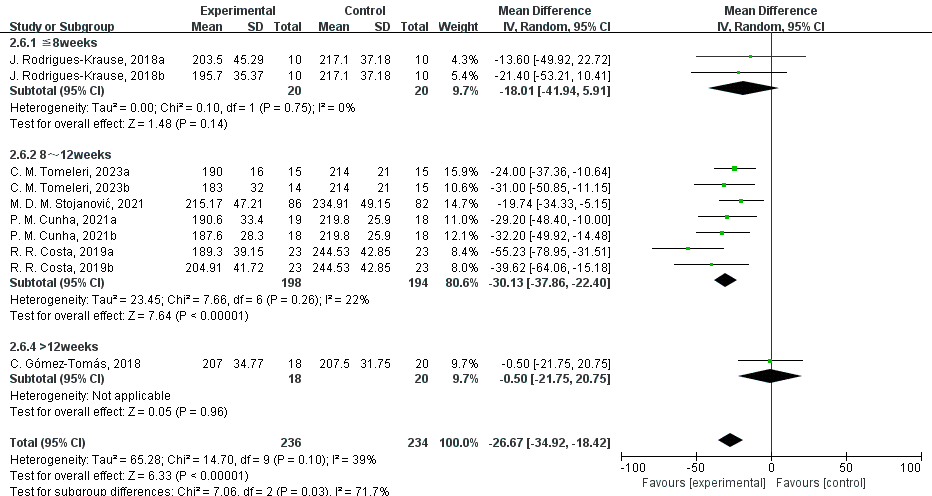


HDL-C


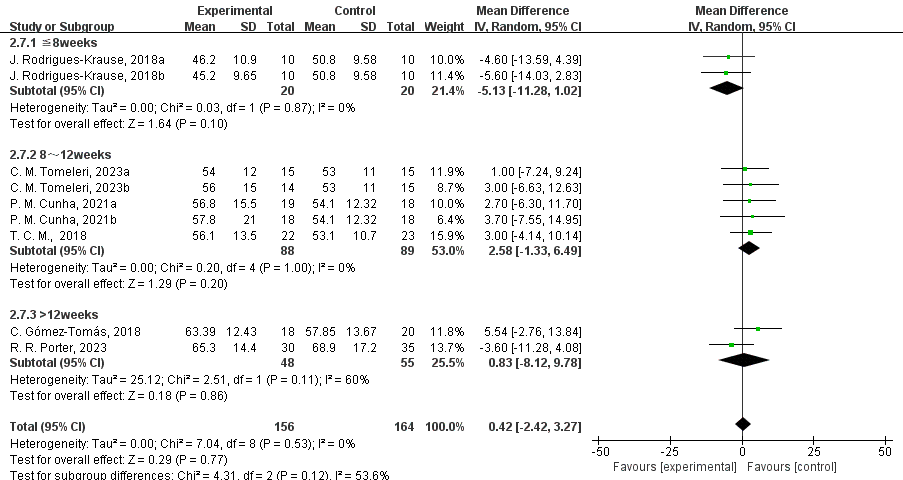


LDL


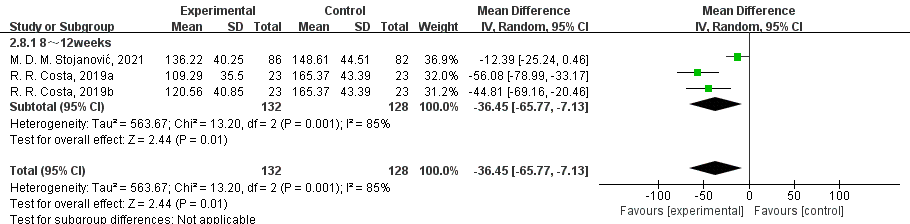


LDL-C


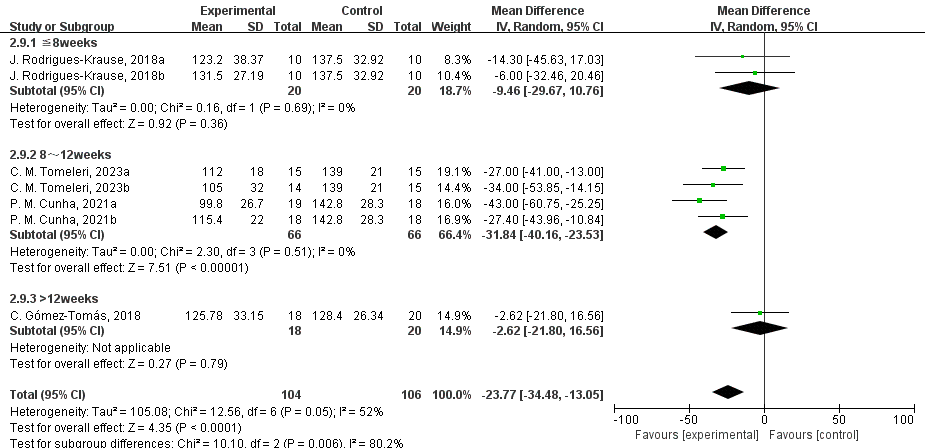


Glu


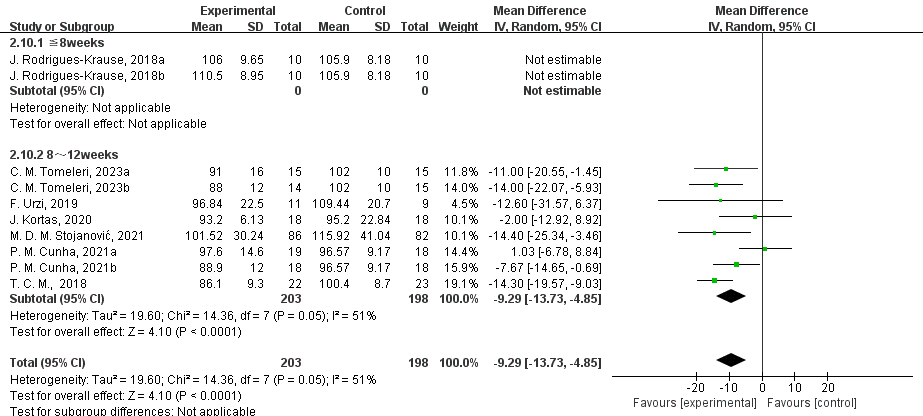


CRP


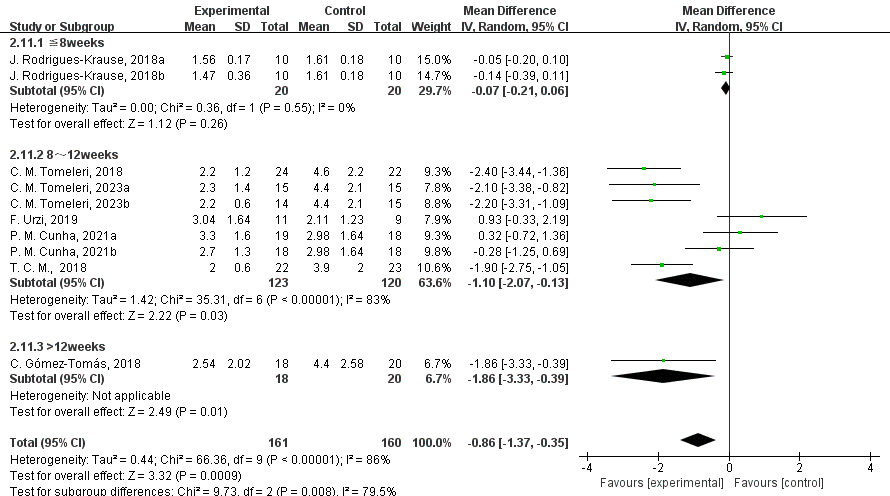


Insulin


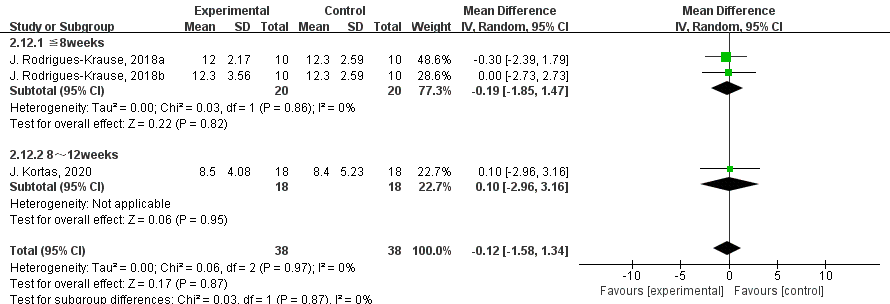


TFM


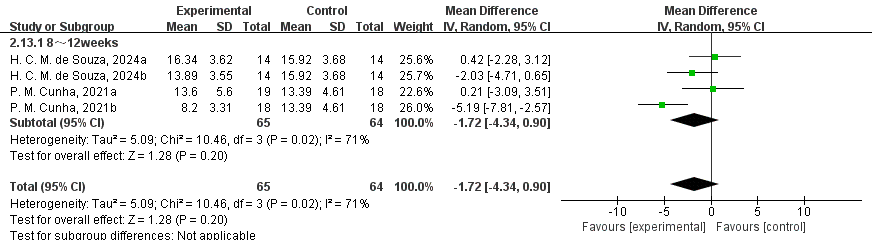


RF


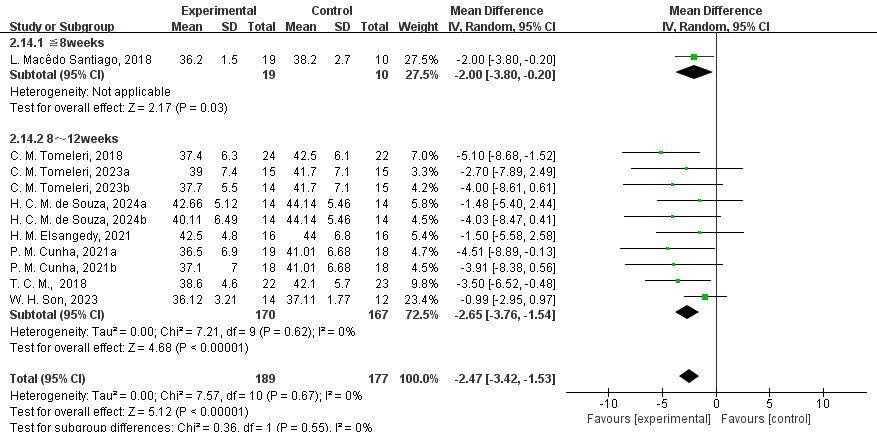


Weight


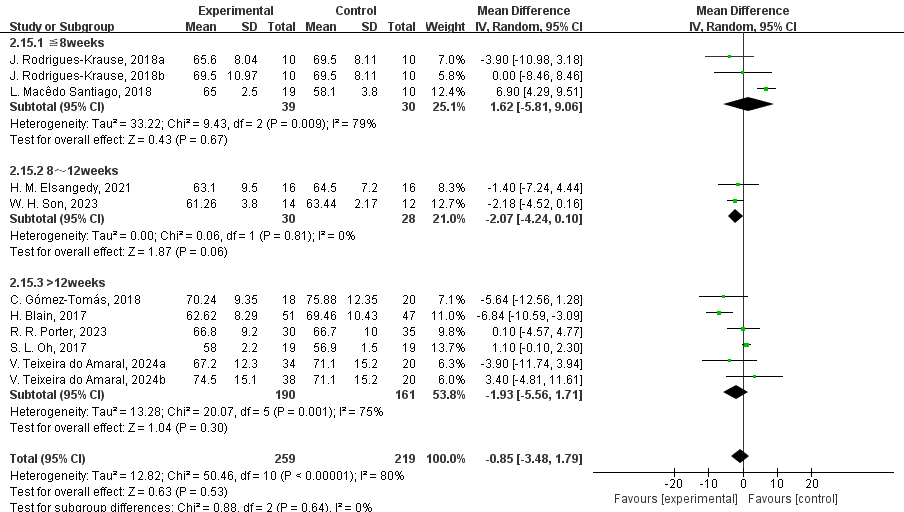


WC


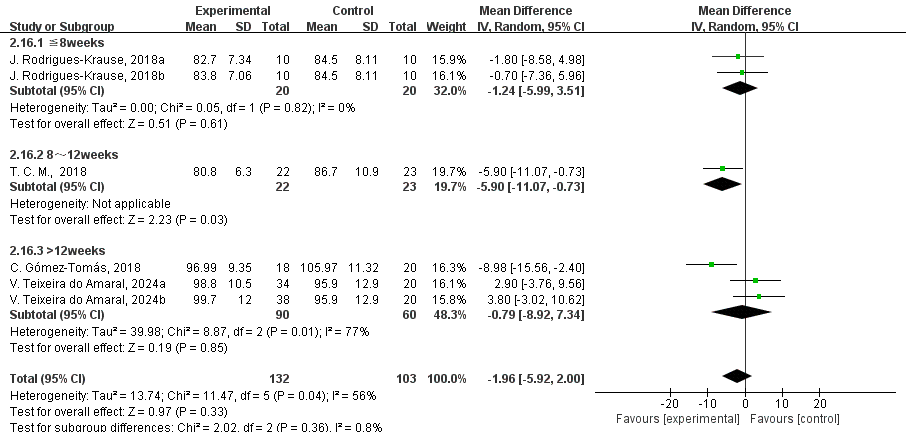


FFM


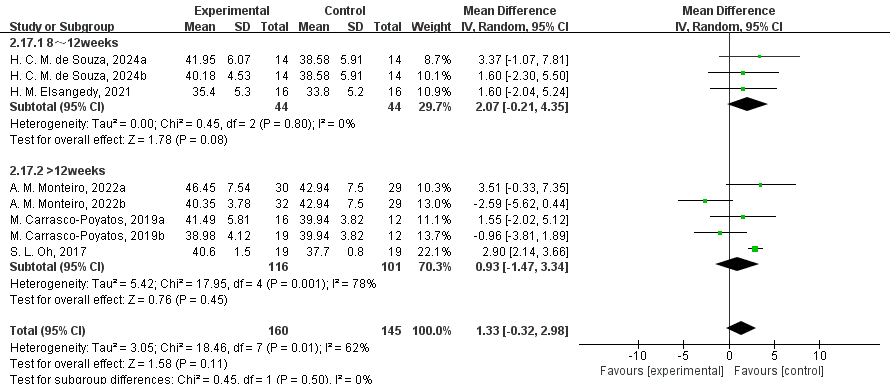


SMM


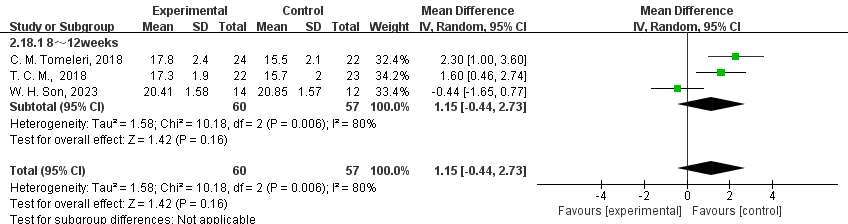


Fig S2. Risk of bias in Randomized Trials


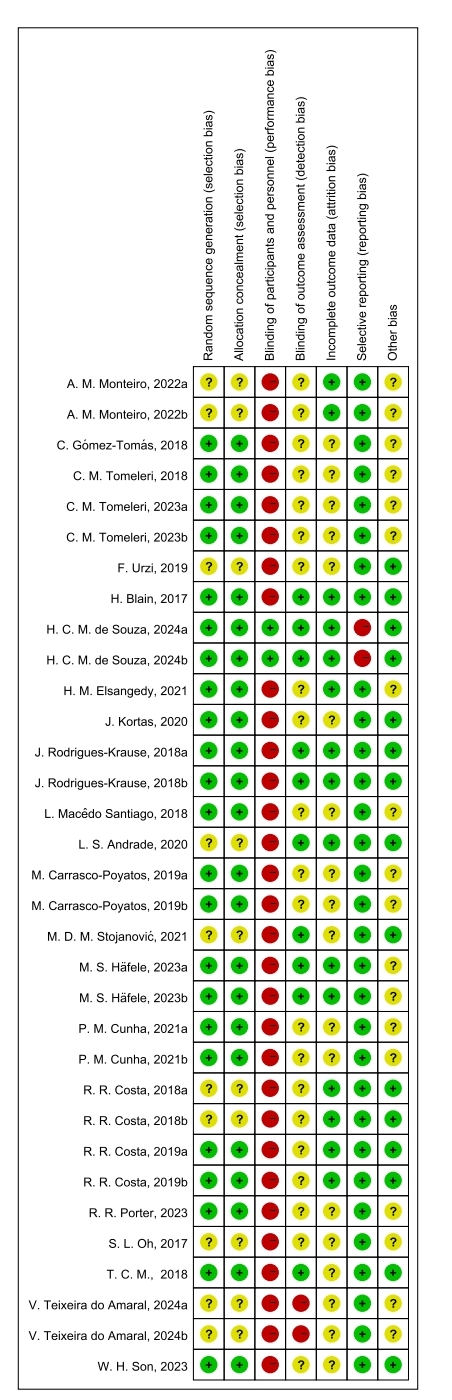


Fig S3. Risk of Bias Graph


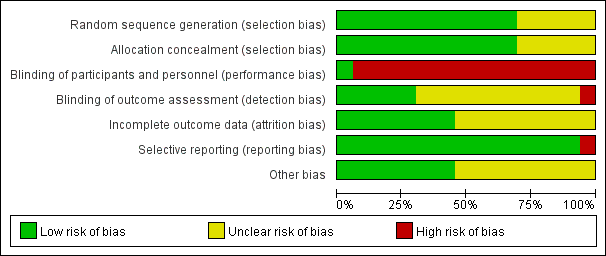

Supplement: Supplementary Material — Supplementary_Figureclean [file RSSN_A_2675444_SM0336.docx]
