# Supplementary material for: Effects of exercise on metabolic risk, cardiovascular fitness, and body composition in elderly women of the past decade: a systematic review and meta-analysis
Source: J Int Soc Sports Nutr. 2026 Jun 2;23(1):2675444. doi: 10.1080/15502783.2026.2675444 (PMC13231817; doi:10.1080/15502783.2026.2675444)
Supplement: Supplementary Material — Supplementary_Tabless.docx [file RSSN_A_2675444_SM0343.docx]

**Supplementary Table**

**Supplementary Table S1** Search Strategies

| Databases | Search | Query | No. of Results |
| --- | --- | --- | --- |
| PubMed  Search Date:  November 2, 2024  Final Search: #12 | #1 | (older women[Title/Abstract]) OR (older females[Title/Abstract]) OR (aged women[Title/Abstract]) OR (elderly women  [Title/Abstract]) OR (elderly females[Title/Abstract]) OR  (postmenopausal women[Title/Abstract]) OR (geriatric women  [Title/Abstract]) | 77,139 |
|  | #2 | exercise[MeSH Terms] | 263,173 |
|  | #3 | (exercise[Title/Abstract]) OR (physical exercise[Title/Abstract])  OR (physical activity[Title/Abstract]) OR (aerobic exercise  [Title/Abstract]) OR (isometric exercise[Title/Abstract]) OR  (acute exercise[Title/Abstract]) OR (exercise training  [Title/Abstract]) OR (biometric exercise[Title/Abstract]) OR  (fitness training[Title/Abstract]) | 470,923 |
|  | #4 | #2 OR #3 | 568,690 |
|  | #5 | resistance training[MeSH Terms] | 13,506 |
|  | #6 | (resistance training[Title/Abstract]) OR (strength training  [Title/Abstract]) OR (weight lifting strengthening program  [Title/Abstract]) OR (resistance exercise[Title/Abstract]) | 24,287 |
|  | #7 | #5 OR #6 | 27,576 |
|  | #8 | #4 OR #7 | 571,837 |
|  | #9 | randomized controlled trials as topic[MeSH Terms] | 179,243 |
|  | #10 | (randomized controlled trials as topic[Title/Abstract]) OR  (clinical trials, randomized[Title/Abstract]) OR  (trials, randomized clinical[Title/Abstract]) OR  (randomized controlled trial[Title/Abstract]) OR  (randomized controlled study[Title/Abstract]) | 139,021 |
|  | #11 | #9 OR #10 | 305,017 |
|  | #12 | #1 AND #8 AND #11 | 425 |
| Cochrane Library  Search Date:  November 2, 2024  Final Search: #12 | #1 | (older women):ab OR (older females):ab OR (aged women):ab OR (elderly women):ab OR (elderly females):ab OR (postmenopausal women):ab OR (geriatric women):ab | 53,680 |
|  | #2 | MeSH descriptor: [Exercise] explode all trees | 39,777 |
|  | #3 | (exercise):ab OR (physical exercise):ab OR  (physical activity):ab OR (aerobic exercise):ab OR  (isometric exercise):ab OR (acute exercise):ab OR  (exercise training):ab OR (biometric exercise):ab OR (fitness training):ab | 140,322 |
|  | #4 | #2 OR #3 | 150,163 |
|  | #5 | MeSH descriptor: [Resistance Training] explode all trees | 5,883 |
|  | #6 | (resistance training):ab OR (strength training):ab OR (weight lifting strengthening program):ab OR (resistance  exercise):ab | 30,110 |
|  | #7 | #5 OR #6 | 30,855 |
|  | #8 | #4 OR #7 | 156,229 |
|  | #9 | MeSH descriptor: [Randomized Controlled Trials as Topic]  explode all trees | 56,210 |
|  | #10 | (randomized controlled trials as topic):ab OR (clinical trials, randomized):ab OR (trials, randomized clinical):ab OR (randomized controlled trial):ab OR (randomized controlled study):ab | 429,503 |
|  | #11 | #9 OR #10 | 458,773 |
|  | #12 | #1 AND #8 AND #11 | 1,833 |
| Web of Science  Search Date:  November 2, 2024  Final Search: #6 | #1 | ((((((AB=(older women)) OR AB=(older females)) OR AB=(aged women)) OR AB=(elderly women)) OR AB=(elderly females)) OR AB=(postmenopausal women)) OR AB=(geriatric women) | [909,789](https://www.webofscience.com/wos/woscc/summary/73d7d4bb-662f-4e53-a00f-b60b4ab1af7e-011d743e3f/relevance/1) |
|  | #2 | ((((((((AB=(exercise)) OR AB=(physical exercise)) OR AB=(physical activity)) OR AB=(aerobic exercise)) OR AB=(isometric exercise)) OR AB=(acute exercise)) OR AB=(exercise training)) OR AB=(biometric exercise)) OR AB=(fitness training) | [684,837](https://www.webofscience.com/wos/woscc/summary/9a315c13-964a-45c9-8156-f52b6b30469d-011d744a12/relevance/1) |
|  | #3 | (((AB=(resistance training)) OR AB=(strength training)) OR AB=(weight lifting strengthening program)) OR AB=(resistance exercise) | [90,470](https://www.webofscience.com/wos/woscc/summary/a802f6b4-2e25-4e19-955e-a53c12f92540-011d7458dc/relevance/1) |
|  | #4 | #2 OR #3 | [733,161](https://www.webofscience.com/wos/woscc/summary/22c893a6-3a00-49dd-a629-364ea73f8225-011d745f93/relevance/1) |
|  | #5 | ((((AB=(randomized controlled trials as topic)) OR AB=(clinical trials, randomized)) OR AB=(trials, randomized clinical)) OR AB=(randomized controlled trial)) OR AB=(randomized controlled study) | [536,656](https://www.webofscience.com/wos/woscc/summary/3d2cc637-05f1-4b4d-94d0-677a8d2b78cc-011d746725/relevance/1) |
|  | #6 | #1 AND #4 AND #5 | [3,044](https://www.webofscience.com/wos/woscc/summary/c058d3f6-2f21-4496-800b-d96888df20bf-011d747f78/relevance/1) |
| Embase  Search Date:  November 2, 2024  Final Search: #12 | #1 | 'older women':ti,ab OR 'older females':ti,ab OR 'aged  women':ti,ab OR 'elderly women':ti,ab OR 'elderly females':ti,ab OR 'postmenopausal women':ti,ab OR 'geriatric women':ti,ab | 105,725 |
|  | #2 | 'exercise'/exp | 480,569 |
|  | #3 | 'exercise':ti,ab OR 'physical exercise':ti,ab OR  'physical activity':ti,ab OR 'aerobic exercise':ti,ab OR 'isometric exercise':ti,ab OR 'acute exercise':ti,ab OR 'exercise training  ':ti,ab OR 'biometric exercise':ti,ab OR 'fitness training':ti,ab | 616,904 |
|  | #4 | #2 OR #3 | 780,358 |
|  | #5 | 'resistance training'/exp | 31,407 |
|  | #6 | 'resistance training':ti,ab OR 'strength training':ti,ab  OR 'weight lifting strengthening program':ti,ab 'resistance exercise':ti,ab | 2,425 |
|  | #7 | #5 OR #6 | 31,915 |
|  | #8 | #4 OR #7 | 780,358 |
|  | #9 | 'randomized controlled trial'/exp | 850,725 |
|  | #10 | 'randomized controlled trials as topic':ti,ab OR 'clinical trials, randomized':ti,ab OR 'trials, randomized clinical':ti,ab  OR 'randomized controlled trial':ti,ab OR  'randomized controlled study':ti,ab | 169,738 |
|  | #11 | #9 OR #10 | 884,929 |
|  | #12 | #1 AND #8 AND #11 | 1,176 |
| CNKI  Search Date:  November 2, 2024  Final Search: #6 | #1 | TKA=('老年女性'+'老年妇女'+'老年女性患者') | 11,225 |
|  | #2 | TKA=('运动'+'运动训练'+'锻炼'+'身体锻炼'+' 功能锻炼'+'运动锻炼'+'实践锻炼'+'康复锻炼'+'身体活动'+'体育锻炼'+'体育运动'+'急性运动'+'静力锻炼'+'等距离运动'+'需氧运动'+'体力活动'+'运动疗法'+'运动, 等长') | 2,059,014 |
|  | #3 | TKA=('抗阻力'+'抗阻力训练'+'抗阻力运动'+'抗阻力量训练'+'抗阻力练习'+'抗阻力跑'+'抗阻力锻炼'+'抗阻力伸腕'+'抗阻力量练习'+'抗阻力功能锻炼'+'等长抗阻力运动') | 1,251 |
|  | #4 | #2 OR #3 | 2,059,014 |
|  | #5 | TKA=('随机对照'+'随机对照试验'+'随机对照研究'+'随机对照临床试验'+'随机对照试验研究'+'随机对照实验'+'随机对照方法'+'随机对照临床研究'+'随机对照法'+'对照临床试验, 随机'+'临床试验, 随机') | 105,766 |
|  | #6 | #1 AND #4 AND #5 | 11 |
| VIP  Search Date:  November 2, 2024  Final Search: #6 | #1 | T=(老年女性 OR 老年妇女 OR 老年女性患者) OR R=(老年女性 OR 老年妇女 OR 老年女性患者) | 29,270 |
|  | #2 | T=(运动 OR 运动训练 OR 锻炼 OR 身体锻炼 OR 功能锻炼  OR 运动锻炼 OR 实践锻炼 OR 康复锻炼 OR 身体活动 OR 体育锻炼 OR 体育运动 OR 急性运动 OR 静力锻炼 OR 等距离运动 OR 需氧运动 OR 体力活动 OR 运动疗法 OR 运动, 等长) OR R=(运动 OR 运动训练 OR 锻炼 OR 身体锻炼 OR 功能锻炼 OR 运动锻炼 OR 实践锻炼 OR 康复锻炼 OR 身体活动 OR 体育锻炼 OR 体育运动 OR 急性运动 OR 静力锻炼 OR 等距离运动 OR 需氧运动 OR 体力活动 OR 运动疗法 OR 运动, 等长) | 1,544,985 |
|  | #3 | T=(抗阻力 OR 抗阻力训练 OR 抗阻力运动 OR 抗阻力量训练 OR 抗阻力练习 OR 抗阻力跑 OR 抗阻力锻炼 OR 抗阻力伸腕 OR 抗阻力量练习 OR 抗阻力功能锻炼 OR 等长抗阻力运动) OR R=(抗阻力 OR 抗阻力训练 OR 抗阻力运动 OR 抗阻力量训练 OR 抗阻力练习 OR 抗阻力跑 OR 抗阻力锻炼 OR 抗阻力伸腕 OR 抗阻力量练习 OR 抗阻力功能锻炼 OR 等长抗阻力运动) | 3,344 |
|  | #4 | #2 OR #3 | 1,547,509 |
|  | #5 | T=(随机对照 OR 随机对照试验 OR 随机对照研究 OR 随机对照临床试验 OR 随机对照试验研究 OR 随机对照实验 OR 随机对照方法 OR 随机对照临床研究 OR 随机对照法 OR 对照临床试验, 随机 OR 临床试验, 随机) OR R=(随机对照 OR 随机对照试验 OR 随机对照研究 OR 随机对照临床试验 OR 随机对照试验研究 OR 随机对照实验 OR 随机对照方法 OR 随机对照临床研究 OR 随机对照法 OR 对照临床试验, 随机 OR 临床试验, 随机) | 1,061,900 |
|  | #6 | #1 AND #4 AND #5 | 67 |
| Wanfang  Search Date:  November 2, 2024  Final Search: #6 | #1 | 主题:(老年女性) or 主题:(老年妇女) or 主题:(老年女性患者) | 65,041 |
|  | #2 | 主题:(运动) or 主题:(运动训练) or 主题:(锻炼) or 主题:(身体锻炼) or 主题:(功能锻炼) or 主题:(运动锻炼) or 主题:(实践锻炼) or 主题:(康复锻炼) or 主题:(身体活动) or 主题:(体育锻炼) or 主题:(体育运动) or 主题:(急性运动) or 主题:(静力锻炼) or 主题:(等距离运动) or 主题:(需氧运动) or 主题:(体力活动) or 主题:(运动疗法) or 主题:(运动, 等长) | 2,317,667 |
|  | #3 | 主题:(抗阻力) or 主题:(抗阻力训练) or 主题:(抗阻力运动) or 主题:(抗阻力量训练) or 主题:(抗阻力练习) or 主题:(抗阻力跑) or 主题:(抗阻力锻炼) or 主题:(抗阻力伸腕) or 主题:(抗阻力量练习) or 主题:(抗阻力功能锻炼) or 主题:(等长抗阻力运动) | 19,626 |
|  | #4 | #2 OR #3 | 2,334,390 |
|  | #5 | 主题:(随机对照) or 主题:(随机对照试验) or 主题:(随机对照研究) or 主题:(随机对照临床试验) or 主题:(随机对照试验研究) or 主题:(随机对照实验) or 主题:(随机对照方法) or 主题:(随机对照临床研究) or 主题:(随机对照法) or 主题:(对照临床试验, 随机) or 主题:(临床试验, 随机) | 2,381,152 |
|  | #6 | #1 AND #4 AND #5 | 467 |
| Sinomed  Search Date:  November 2, 2024  Final Search: #6 | #1 | ["老年女性"[常用字段:智能] OR "老年妇女"[常用字段:智能] OR "老年女性患者"[常用字段:智能]](javascript:toDoRelimitSearch();) | 10,052 |
|  | #2 | "运动"[常用字段:智能] OR "运动训练"[常用字段:智能] OR "锻炼"[常用字段:智能] OR "身体锻炼"[常用字段:智能] OR "功能锻炼 "[常用字段:智能] OR "运动锻炼"[常用字段:智能] OR "实践锻炼"[常用字段:智能] OR "康复锻炼"[常用字段:智能] OR "身体活动"[常用字段:智能] OR "体育锻炼"[常用字段:智能] OR "体育运动"[常用字段:智能] OR "急性运动"[常用字段:智能] OR "静力锻炼"[常用字段:智能] OR "等距离运动"[常用字段:智能] OR "需氧运动"[常用字段:智能] OR "体力活动"[常用字段:智能] OR "运动疗法"[常用字段:智能] OR "运动, 等长"[常用字段:智能] | [446,334](javascript:historyLink(') |
|  | #3 | ["抗阻力"[常用字段:智能] OR "抗阻力训练"[常用字段:智能] OR "抗阻力运动"[常用字段:智能] OR "抗阻力量训练"[常用字段:智能] OR "抗阻力练习"[常用字段:智能] OR "抗阻力跑"[常用字段:智能] OR "抗阻力锻炼"[常用字段:智能] OR "抗阻力伸腕"[常用字段:智能] OR "抗阻力量练习"[常用字段:智能] OR "抗阻力功能锻炼"[常用字段:智能] OR "等长抗阻力运动"[常用字段:智能]](javascript:toDoRelimitSearch();) | 385 |
|  | #4 | #2 OR #3 | 446,393 |
|  | #5 | "随机对照"[常用字段:智能] OR "随机对照试验"[常用字段:智能] OR "随机对照研究"[常用字段:智能] OR "随机对照临床试验"[常用字段:智能] OR "随机对照试验研究"[常用字段:智能] OR "随机对照实验"[常用字段:智能] OR "随机对照方法"[常用字段:智能] OR "随机对照临床研究"[常用字段:智能] OR "随机对照法"[常用字段:智能] OR "对照临床试验, 随机"[常用字段:智能] OR "临床试验, 随机"[常用字段:智能] | [236,840](javascript:historyLink(') |
|  | #6 | #1 AND #4 AND #5 | 10 |

**Supplementary Table S2** Intervention characteristics

| First Author, Year | Group | Intensity | Supervision | Adherence (%) |
| --- | --- | --- | --- | --- |
| Rodrigues-Krause, 2018 | Dance Group | ~60% VO₂peak (controlled by music BPM) | Fully Supervised | >95% |
|  | Walking Group | 50-65% VO₂peak (based on HR) | Fully Supervised | >95% |
|  | CG | Low intensity, without discomfort | Fully Supervised | >95% |
| H. C. M. de Souza,2024 | IMT + WBV | IMT: 40% of MIP, weekly adjustment; | IMT: Partially supervised (one series with therapist, remaining sessions at home) | Not Reported |
|  |  | WBV: 35 Hz, amplitude 2-4 mm | WBV: Fully supervised |  |
|  | IMTsham + WBV | IMT: Fixed load of 10 cmH₂O; | IMT: Partially supervised (one series with therapist, remaining sessions at home) | Not Reported |
|  |  | WBV: 35 Hz, amplitude 2-4 mm | WBV: Fully supervised |  |
|  | IMTsham + WBVsham | IMT: Fixed load of 10 cmH₂O; | IMT: Partially supervised (one series with therapist, remaining sessions at home) | Not Reported |
|  |  | WBV: Sham (minimal frequency, no therapeutic effect) | WBV: Fully supervised (positioned on platform) |  |
| C. M. Tomeleri, 2018 | EG | 3 sets of 10-15 RM; loads adjusted weekly | Fully supervised by experienced physical education professionals | ≥ 85% |
|  | CG | No exercise | Unsupervised | Not Reported |
| L. Macêdo Santiago, 2018 | EG | 8-12 MR; loads progressively increased; based on Borg Scale | Fully supervised by one physiotherapist and two physical education professionals | > 85% |
|  | CG | No exercise | Unsupervised | Not Reported |
| H. M. Elsangedy, 2021 | SSRT | Self-Selected (~50% of 1RM, Low-to-Moderate) | Fully supervised: Individual supervision by physical education professionals) | 100% |
|  | CG | No exercise | Supervised board games and manual activities | 100% |
| M. Carrasco-Poyatos, 2019 | Pilates Exercise Program | Moderate to Vigorous | Fully supervised: Same certified instructor for both interventions | 80% |
|  | Muscular Exercise Program | Moderate to Vigorous | Fully supervised: Same certified instructor for both interventions | 95% |
|  | CG | Normal physical activity habits | Encouraged to maintain habits | 60% |
| M. D. M. Stojanović, 2021 | Elastic Band Training | Low-Load (40-60% of 1RM, OMNI-RES 4-5) | Fully supervised, conducted in groups, monitored by 2 qualified instructors | 92% |
|  | CG | Institution-based activities | Encouraged to maintain normal institution activities | Not Reported |
| C. Gómez-Tomás, 2018 | Intervention Group | Progressive | Fully supervised, monitored by the principal investigator | Not Reported |
|  | CG | No exercise | Unsupervised | Not Reported |
| R. R. Costa, 2018 | WA | 80-100% of Heart Rate at Anaerobic Threshold; Linear periodization. | Fully supervised by the same instructor | >95% |
|  | WR | Maximal effort/velocity per repetition to maximize hydrodynamic resistance | Fully supervised by the same instructor | >95% |
|  | CG | Low intensity; instructed to move slowly to avoid water resistance | Fully supervised to ensure compliance with low-intensity instructions and prevent any training stimuli | >95% |
| F. Urzi, 2019 | EG | Moderate intensity, controlled using Borg's Rate of Perceived Exertion (RPE) level 12-14 ("somewhat hard"). The elastic band resistance remained the same throughout the 12-week intervention. | Sessions were conducted in the nursing home hall in a supervised setting by the same instructor to ensure intensity control and proper exercise form. | Average 87.6% (±8.8%) |
|  | GG | No any placebo or treatment | Unsupervised | Not Reported |
| P. M. Cunha, 2021 | LV | 10-15 RM for all exercises. | Each participant was individually supervised by physical education professionals | >85% |
|  | HV | 10-15 RM for all exercises. | Each participant was individually supervised by physical education professionals | >85% |
|  | CG | No exercise | Unsupervised | Not Reported |
| J. Kortas, 2020 | NW | 70% of individual maximum ability (HRmax: 60-70%) | Polar M200 heart rate monitors used; technique instruction and weekly supervision | < 80% |
|  | CG | No exercise | Unsupervised | Not Reported |
| A. M. Monteiro, 2022 | Group A (Aerobic First) | Aerobic: 12-14 on Borg's RPE scale (Moderate). Resistance: Progressive (started with 1 set of 8 reps, progressed to 3 sets of 12-15 reps). | The exercise program and evaluations were applied by the researcher | >75% |
|  | Group B (Resistance First) | Aerobic: 12-14 on Borg's RPE scale (Moderate). Resistance: Progressive (started with 1 set of 8 reps, progressed to 3 sets of 12-15 reps). | The exercise program and evaluations were applied by the researcher | >75% |
|  | GC | no exercise | Unsupervised | Not Reported |
| S. L. Oh, 2017 | TG | Thera-Band resistance training based on the 10-20 RM principle. | First 8 weeks were supervised training by expert trainers. The following 10 weeks were self-directed training, supported by a guidebook, self-check list, and DVD demonstrations. | 94.2% |
|  | GC | Low-intensity stretching (static and dynamic) program, once per week for 1 hour. | Conducted under supervision | Not Reported |
| R. R. Porter, 2023 | Moderate Dose | 50–55% heart rate reserve | All exercise sessions were supervised in a clinical exercise research setting. | 98.90% |
|  | Low Dose | 50–55% heart rate reserve | All exercise sessions were supervised in a clinical exercise research setting. | 100.00% |
| H. Blain, 2017 | EG | 60-80% of maximum heart rate | 2 supervised + 1 unsupervised session/week | 92% |
|  | CG | No exercise | Unsupervised | Not Reported |
| T. C. M., 2018 | EG | 3 sets of 10-15 repetition maximum (RM) | All sessions were personally supervised by physical education professionals with substantial resistance training experience | >85% |
|  | CG | No exercise | Unsupervised | Not Reported |
| W. H. Son, 2023 | EG | Moderate (64–76% HRmax, ~100 steps/min, 7000-9999 steps/day) | Monitored (via Fitbit and HR monitor) | 100% |
|  | CG | Not Reported | Unsupervised | Not Reported |
| C. M. Tomeleri, 2023 | MJ-SJ | 3 sets of 10-15 RM | By physical education professionals | 98.80% |
|  | SJ-MJ | 3 sets of 10-15 RM | Yes, by physical education professionals | 96.30% |
|  | CG | No exercise | Unsupervised | Not Reported |
| M. S. Häfele, 2023 | AT | Prescribed based on the heart rate percentage at the anaerobic threshold | By two experienced instructors | 83 ± 7% |
|  | AT-CT | Aerobic part: Heart Rate at Anaerobic Threshold; Resistance part: Maximal effort | By two experienced instructors | 89 ± 11% |
|  | CG | Performed as slowly as possible | By instructors | 82 ± 10% |
| V. Teixeira do Amaral, 2024 | HIIT + RT | High Intensity: The HIIT protocol involved cycles of high-intensity exercise on a cycle ergometer interspersed with active rest. | Sessions were held twice weekly and were designed and overseen by the research team. | Not Reported |
|  | MICT + RT | Moderate Intensity: The MICT protocol involved continuous exercise on a cycle ergometer at 60-70% of heart rate reserve. | Sessions were held twice weekly and were designed and overseen by the research team. | Not Reported |
|  | RT | Community-based exercise programs | Sessions were held twice weekly and were designed and overseen by the research team. | Not Reported |

Abbreviations: AT: Aerobic Training; CG: Control Group; cmH₂O: Centimeters of Water; CT: Combined Training; HIIT+RT: High-Intensity Interval Training combined with Resistance Training

;Hz: Hertz; HV: High-Volume; IMT: Inspiratory Muscle Training; IMTsham: Sham training for IMT; LV: Low-Volume; MICT+RT: Moderate Intensity Continuous Training combined with Resistance Training; MIP: Maximal Inspiratory Pressure; MJ: Multi-joint; NW: Nordic Walking; RM: Repetition Maximum; RT: Resistance Training; SJ: Single-joint; SSRT: Self-Selected Resistance Training; TG: Training Group; WA: Water-based Aerobic; WBV: Whole-Body Vibration; WBVsham: Sham training for WBV; WR: Water-based Resistance

**Supplementary Table S3** Sensitivity Analyses

| Number of  studies | | Outcomes | Before sensitivity analyses | | | Method  of sensitivity analyses | Number of  studies | After sensitivity analyses | | |
| --- | --- | --- | --- | --- | --- | --- | --- | --- | --- | --- |
|  |  | | MD 95% CI | I2 | P |  |  | MD 95% CI | I2 | P |
| Sensitivity of Cardiovascular fitness | | | | | | | | | |  |
| 2 | HRmax | | -0.29  [-5.90, 5.33] | 0% | 0.92 | – | 2 | -0.29  [-5.90, 5.33] | 0% | 0.92 |
| 5 | VO2peak | | 2.78  [1.87, 3.70] | 0% | <0.00001 | – | 5 | 2.78  [1.87, 3.70] | 0% | <0.00001 |
| 2 | SBP | | -8.35  [-13.71, -2.99] | 16% | 0.002 | Removing  M. S. Häfele, 2023a | 2 | -9.96  [-15.17, -4.75] | 1% | 0.0002 |
| 2 | DBP | | -3.26  [-6.46, -0.07] | 0% | 0.05 | – | 2 | -3.26  [-6.46, -0.07] | 0% | 0.05 |
| Sensitivity of metabolic risk | | | | | | | | | | |
| 8 | TG | | -8.56  [-16.72, -0.40] | 10% | 0.04 | Removing  R. R. Porter, 2023 | 7 | -11.03  [-18.97, -3.10] | 0% | 0.006 |
| 6 | TC | | -26.67  [-34.92, -18.42] | 39% | <0.00001 | Removing  C. Gómez-Tomás, 2018  & R. R. Costa, 2019a | 5 | -26.42  [-33.14, -19.70] | 0% | <0.00001 |
| 6 | HDL-C | | 0.42  [-2.42, 3.27] | 0% | 0.77 | – | 6 | 0.42  [-2.42, 3.27] | 0% | 0.77 |
| 2 | LDL | | -36.45  [-65.77, -7.13] | 85% | 0.01 | Removing  M. D. M. Stojanović, 2021 | 1 | -50.79  [-67.48, -34.10] | 0% | <0.00001 |
| 4 | LDL-C | | -23.77  [-34.48, -13.05] | 52% | <0.0001 | Removing  C. Gómez-Tomás, 2018  & P. M. Cunha, 2021a | 3 | -25.28  [-33.81, -16.74] | 0% | <0.00001 |
| 7 | Glu | | -6.67 [-11.59, -1.75] | 70% | 0.008 | Removing   \| C. M. Tomeleri, 2023a \| \| --- \| \| &C. M. Tomeleri, 2023b  &J. Rodrigues-Krause, 2018a  &J. Rodrigues-Krause, 2018b  &T. C. M., 2018   \|  \| \| --- \| \| | 4 | -5.54 [-9.75, -1.33] | 39% | 0.01 |
| 7 | CRP | | -0.86  [-1.37, -0.35] | 86% | 0.0009 | Removing  T. C. M., 2018  & C. M. Tomeleri, 2018 | 5 | -0.47  [-0.92, -0.03] | 78% | 0.04 |
| 2 | Insulin | | -0.12  [-1.58, 1.34] | 0% | 0.87 | – | 2 | -0.12  [-1.58, 1.34] | 0% | 0.87 |
| Sensitivity of body composition | | | | | | | | | |  |
| 2 | TFM | | -1.72  [-4.34, 0.90] | 71% | 0.20 | Removing  P. M. Cunha, 2021b | 2 | -0.56  [-2.21, 1.09] | 0% | 0.51 |
| 8 | RF | | -2.47  [-3.42, -1.53] | 0% | <0.00001 | – | 8 | -2.47  [-3.42, -1.53] | 0% | <0.00001 |
| 9 | Weight | | -0.85  [-3.48, 1.79] | 80% | 0.53 | Removing  C. Gómez-Tomás, 2018  & H. Blain, 2017  & L. Macêdo Santiago, 2018  & W. H. Son, 2023 | 5 | 0.76  [-0.33, 1.86] | 0% | 0.17 |
| 4 | WC | | -1.96  [-5.92, 2.00] | 56% | 0.33 | Removing  C. Gómez-Tomás, 2018  & T. C. M., 2018 | 2 | 1.04  [-2.32, 4.41] | 0% | 0.54 |
| 5 | FFM | | 1.33  [-0.32, 2.98] | 62% | 0.11 | Removing  A. M. Monteiro, 2022b  & S. L. Oh, 2017 | 4 | 1.38  [-0.09, 2.85] | 0% | 0.07 |
| 3 | SMM | | 1.15  [-0.44, 2.73] | 80% | 0.16 | Removing  W. H. Son, 2023 | 2 | 1.90  [1.05, 2.76] | 0% | <0.0001 |

Abbreviations: CRP = C-reactive protein; DBP = Diastolic blood pressure; FFM = Fat-free mass; Glu = Glucose; HDL-C = High-density lipoprotein cholesterol; HRmax = Maximal heart rate; LDL = Low-density lipoprotein; LDL-C = Low-density lipoprotein cholesterol; MD = Mean Difference; RF = Relative body fat; SBP = Systolic blood pressure; SSM = Skeletal muscle mass; TC = Total cholesterol; TFM = Total fat mass; TG = Triglycerides; VO₂peak = Maximal oxygen uptake; WC = Waist circumfere

**Supplementary Table S4** GRADE Quality Assessment

| Outcome | Quality assessment | | | | | | Quality* |
| --- | --- | --- | --- | --- | --- | --- | --- |
|  | No of studies | Risk of bias | Inconsistency | Indirectness | Imprecision | Other considerations |  |
| HRmax | 3 | not serious | not serious | not serious | very serious | none | ㊉㊉㊀㊀ LOW |
| VO2peak | 7 | not serious | not serious | not serious | serious | none | ㊉㊉㊉㊀ MODERATE |
| SBP | 3 | not serious | not serious | not serious | serious | none | ㊉㊉㊉㊀ MODERATE |
| DBP | 3 | not serious | not serious | not serious | very serious | none | ㊉㊉㊀㊀ LOW |
| TG | 12 | not serious | not serious | not serious | serious | none | ㊉㊉㊉㊀ MODERATE |
| TC | 10 | not serious | not serious | not serious | serious | none | ㊉㊉㊉㊀ MODERATE |
| HDL-C | 9 | not serious | not serious | not serious | very serious | none | ㊉㊉㊀㊀ LOW |
| LDL | 3 | not serious | very serious | not serious | serious | none | ㊉㊀㊀㊀ VERY LOW |
| LDL-C | 7 | not serious | serious | not serious | serious | none | ㊉㊉㊀㊀ LOW |
| Glu | 10 | not serious | very serious | not serious | serious | none | ㊉㊀㊀㊀ VERY LOW |
| CRP | 10 | not serious | very serious | not serious | serious | none | ㊉㊀㊀㊀ VERY LOW |
| Insulin | 3 | not serious | not serious | not serious | very serious | none | ㊉㊉㊀㊀ LOW |
| TFM | 4 | not serious | serious | not serious | very serious | none | ㊉㊀㊀㊀ VERY LOW |
| RF | 11 | not serious | not serious | not serious | serious | none | ㊉㊉㊉㊀ MODERATE |
| Weight | 11 | not serious | very serious | not serious | serious | none | ㊉㊀㊀㊀ VERY LOW |
| WC | 6 | serious | serious | not serious | very serious | none | ㊉㊀㊀㊀ VERY LOW |
| FFM | 8 | serious | serious | not serious | very serious | none | ㊉㊀㊀㊀ VERY LOW |
| SMM | 3 | not serious | very serious | not serious | very serious | none | ㊉㊀㊀㊀ VERY LOW |
| *Certainty of evidence according to Grading of Recommendations Assessment, Development, and Evaluations (GRADE):  High: We are very confident in the estimated effect  Moderate: Our confidence in the estimated effect is moderate  Low: We have limited confidence in the estimated effect  Very low: We have very little confidence in the estimated effect  Not of participants: Total number of participants with pooled effects | | | | | | | |

**Supplementary Table S5** Subgroup Analysis

| Outcomes/subgroup | Number of  studies | Number of  participants | Statistical method | Effect estimate | The value of P | Heterogeneity(I2) |
| --- | --- | --- | --- | --- | --- | --- |
| HRmax |  |  |  |  |  |  |
| 8～12weeks | 1 | 46 | MD (IV, Random, 95% CI) | 0.00 [-9.04, 9.04] | 1 | – |
| >12weeks | 1 | 52 | MD (IV, Random, 95% CI) | -0.46 [-7.62, 6.69] | 0.90 | 0% |
| VO2peak |  |  |  |  |  |  |
| ≦8weeks | 1 | 30 | MD (IV, Random, 95% CI) | 3.41 [1.75, 5.07] | <0.0001 | 0% |
| 8～12weeks | 3 | 133 | MD (IV, Random, 95% CI) | 2.74 [1.50, 3.99] | <0.0001 | 0% |
| >12weeks | 1 | 65 | MD (IV, Random, 95% CI) | 1.70 [-0.62, 4.02] | 0.15 | – |
| SBP |  |  |  |  |  |  |
| 8～12weeks | 1 | 45 | MD (IV, Random, 95% CI) | -11.70 [-17.88, -5.52] | 0.0002 | – |
| >12weeks | 1 | 52 | MD (IV, Random, 95% CI) | -4.48 [-11.86, 2.90] | 0.23 | 0% |
| DBP |  |  |  |  |  |  |
| 8～12weeks | 1 | 45 | MD (IV, Random, 95% CI) | -2.80 [-6.75, 1.15] | 0.16 | – |
| >12weeks | 1 | 52 | MD (IV, Random, 95% CI) | -4.15 [-9.62, 1.31] | 0.14 | 0% |
| TG |  |  |  |  |  |  |
| ≦8weeks | 1 | 30 | MD (IV, Random, 95% CI) | 0.03 [-37.25, 37.31] | 1 | 30% |
| 8～12weeks | 5 | 371 | MD (IV, Random, 95% CI) | -9.55 [-18.71, -0.40] | 0.04 | 0% |
| >12weeks | 2 | 103 | MD (IV, Random, 95% CI) | -3.88 [-39.26, 31.50] | 0.83 | 79% |
| TC |  |  |  |  |  |  |
| ≦8weeks | 1 | 30 | MD (IV, Random, 95% CI) | -18.01 [-41.94, 5.91] | 0.14 | 0% |
| 8～12weeks | 4 | 336 | MD (IV, Random, 95% CI) | -30.13 [-37.86, -22.40] | <0.00001 | 22% |
| >12weeks | 1 | 38 | MD (IV, Random, 95% CI) | -0.50 [-21.75, 20.75] | 0.96 | – |
| HDL-C |  |  |  |  |  |  |
| ≦8weeks | 1 | 30 | MD (IV, Random, 95% CI) | -5.13 [-11.28, 1.02] | 0.10 | 0% |
| 8～12weeks | 3 | 144 | MD (IV, Random, 95% CI) | 2.58 [-1.33, 6.49] | 0.20 | 0% |
| >12weeks | 2 | 103 | MD (IV, Random, 95% CI) | 0.83 [-8.12, 9.78] | 0.86 | 60% |
| LDL |  |  |  |  |  |  |
| 8～12weeks | 2 | 237 | MD (IV, Random, 95% CI) | -36.45 [-65.77, -7.13] | 0.01 | 85% |
| LDL-C |  |  |  |  |  |  |
| ≦8weeks | 1 | 30 | MD (IV, Random, 95% CI) | -9.46 [-29.67, 10.76] | 0.36 | 0% |
| 8～12weeks | 2 | 99 | MD (IV, Random, 95% CI) | -31.84 [-40.16, -23.53] | <0.00001 | 0% |
| >12weeks | 1 | 38 | MD (IV, Random, 95% CI) | -2.62 [-21.80, 16.56] | 0.79 | – |
| Glucose |  |  |  |  |  |  |
| ≦8weeks | 1 | 30 | MD (IV, Random, 95% CI) | 2.45 [-2.98, 7.87] | 0.38 | 0% |
| 8～12weeks | 6 | 368 | MD (IV, Random, 95% CI) | -9.29 [-13.73, -4.85] | <0.0001 | 51% |
| CRP |  |  |  |  |  |  |
| ≦8weeks | 1 | 30 | MD (IV, Random, 95% CI) | -0.07 [-0.21, 0.06] | 0.26 | 0% |
| 8～12weeks | 5 | 210 | MD (IV, Random, 95% CI) | -1.10 [-2.07, -0.13] | 0.03 | 83% |
| >12weeks | 1 | 38 | MD (IV, Random, 95% CI) | -1.86 [-3.33, -0.39] | 0.01 | – |
| Insulin |  |  |  |  |  |  |
| ≦8weeks | 1 | 30 | MD (IV, Random, 95% CI) | -0.19 [-1.85, 1.47] | 0.82 | 0% |
| 8～12weeks | 1 | 36 | MD (IV, Random, 95% CI) | 0.10 [-2.96, 3.16] | 0.95 | – |
| TFM |  |  |  |  |  |  |
| 8～12weeks | 2 | 97 | MD (IV, Random, 95% CI) | -1.72 [-4.34, 0.90] | 0.20 | 71% |
| RF |  |  |  |  |  |  |
| ≦8weeks | 1 | 29 | MD (IV, Random, 95% CI) | -2.00 [-3.80, -0.20] | 0.03 | – |
| 8～12weeks | 7 | 290 | MD (IV, Random, 95% CI) | -2.65 [-3.76, -1.54] | <0.00001 | 0% |
| Weight |  |  |  |  |  |  |
| ≦8weeks | 2 | 59 | MD (IV, Random, 95% CI) | 1.62 [-5.81, 9.06] | 0.67 | 79% |
| 8～12weeks | 2 | 58 | MD (IV, Random, 95% CI) | -2.07 [-4.24, 0.10] | 0.06 | 0% |
| >12weeks | 5 | 331 | MD (IV, Random, 95% CI) | -1.93 [-5.56, 1.71] | 0.30 | 75% |
| WC |  |  |  |  |  |  |
| ≦8weeks | 1 | 30 | MD (IV, Random, 95% CI) | -1.24 [-5.99, 3.51] | 0.61 | 0% |
| 8～12weeks | 1 | 45 | MD (IV, Random, 95% CI) | -5.90 [-11.07, -0.73] | 0.03 | – |
| >12weeks | 2 | 130 | MD (IV, Random, 95% CI) | -0.79 [-8.92, 7.34] | 0.85 | 77% |
| FFM |  |  |  |  |  |  |
| 8～12weeks | 2 | 74 | MD (IV, Random, 95% CI) | 2.07 [-0.21, 4.35] | 0.08 | 0% |
| >12weeks | 3 | 176 | MD (IV, Random, 95% CI) | 0.93 [-1.47, 3.34] | 0.45 | 78% |
| SMM |  |  |  |  |  |  |
| 8～12weeks | 3 | 117 | MD (IV, Random, 95% CI) | 1.15 [-0.44, 2.73] | 0.16 | 80% |

Abbreviations: CRP = C-reactive protein; DBP = Diastolic blood pressure; FFM = Fat-free mass; Glu = Glucose; HDL-C = High-density lipoprotein cholesterol; HRmax = Maximal heart rate; LDL = Low-density lipoprotein; LDL-C = Low-density lipoprotein cholesterol; MD = Mean Difference; RF = Relative body fat; SBP = Systolic blood pressure; SSM = Skeletal muscle mass; TC = Total cholesterol; TFM = Total fat mass; TG = Triglycerides; VO₂peak = Maximal oxygen uptake; WC = Waist circumference
